# Single-molecule tracking reveals the functional allocation, *in vivo* interactions, and spatial organization of universal transcription factor NusG

**DOI:** 10.1016/j.molcel.2024.01.025

**Published:** 2024-02-21

**Authors:** Hafez El Sayyed, Oliver J. Pambos, Mathew Stracy, Max E. Gottesman, Achillefs N. Kapanidis

**Affiliations:** 1 Gene Machines Group, Clarendon Laboratory, Department of Physics, University of Oxford, Oxford, UK; 2 Kavli Institute of Nanoscience Discovery, University of Oxford, Dorothy Crowfoot Hodgkin Building, Oxford, UK; 3 Sir William Dunn School of Pathology, University of Oxford, South Parks Rd, Oxford, UK; 4 Department of Microbiology & Immunology, Columbia University Medical Center, New York, NY, USA

## Abstract

During transcription elongation, NusG aids RNA polymerase by inhibiting pausing, promoting anti-termination on rRNA operons, coupling transcription with translation on mRNA genes, and facilitating Rho-dependent termination. Despite extensive work, the *in vivo* functional allocation and spatial distribution of NusG remain unknown. Using single-molecule tracking and super-resolution imaging in live *E. coli* cells, we found NusG predominantly in a chromosome-associated population (binding to RNA polymerase in elongation complexes) and a slowly diffusing population complexed with the 30S ribosomal subunit; the latter provides a “30S-guided” path for NusG into transcription elongation. Only ~10% of NusG is fast diffusing, with its mobility suggesting non-specific interactions with DNA for >50% of the time. Antibiotic treatments and deletion mutants revealed that chromosome-associated NusG participates mainly in *rrn* anti-termination within phase-separated transcriptional condensates and in transcription-translation coupling. This study illuminates the multiple roles of NusG and offers a guide on dissecting multi-functional machines via *in vivo* imaging.

## Graphical abstract

**Graphical abstract:**
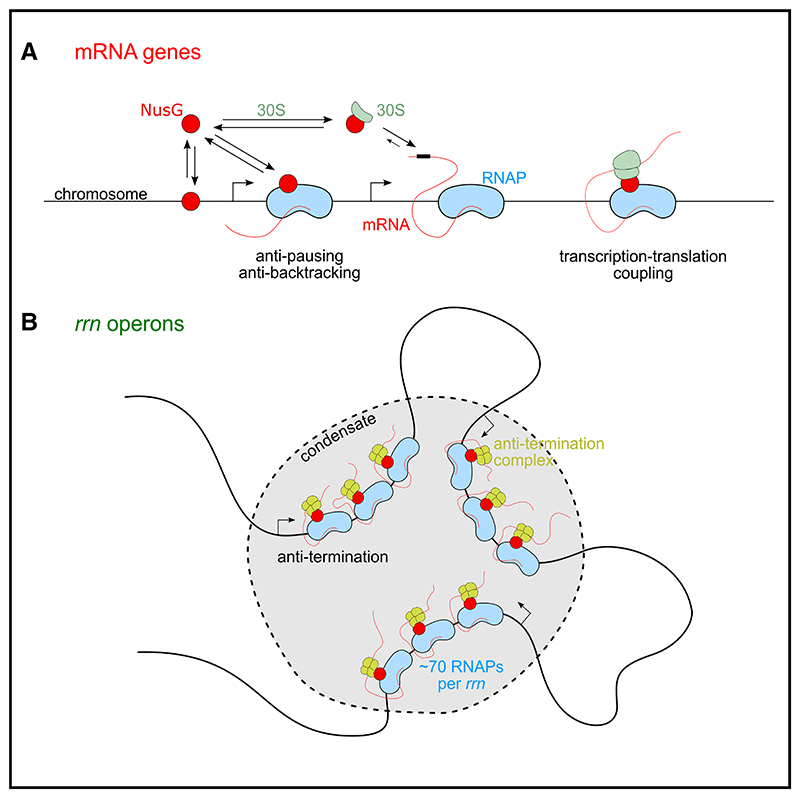

## Introduction

Transcription, a process central to all living organisms, is orchestrated by multi-functional protein machine RNA polymerase (RNAP). After the initial recognition of a promoter region, RNAP initiates RNA synthesis and escapes from the promoter after transcribing up to ~15 nt.^1,2^ RNAP then enters transcription elongation, which continues processively until termination signals are encountered, at which point the nascent RNA and RNAP dissociate from DNA.^3^ During transcription elongation, RNAP encounters DNA sequences and other signals that can slow down or stop transcription via processes such as pausing, backtracking and premature transcription termination. To counteract these impediments, maintain a steady RNA production, and enable additional mechanisms for transcriptional fidelity and regulation, cells employ many elongation factors that interact in different ways with RNAP.

**Figure 1:**
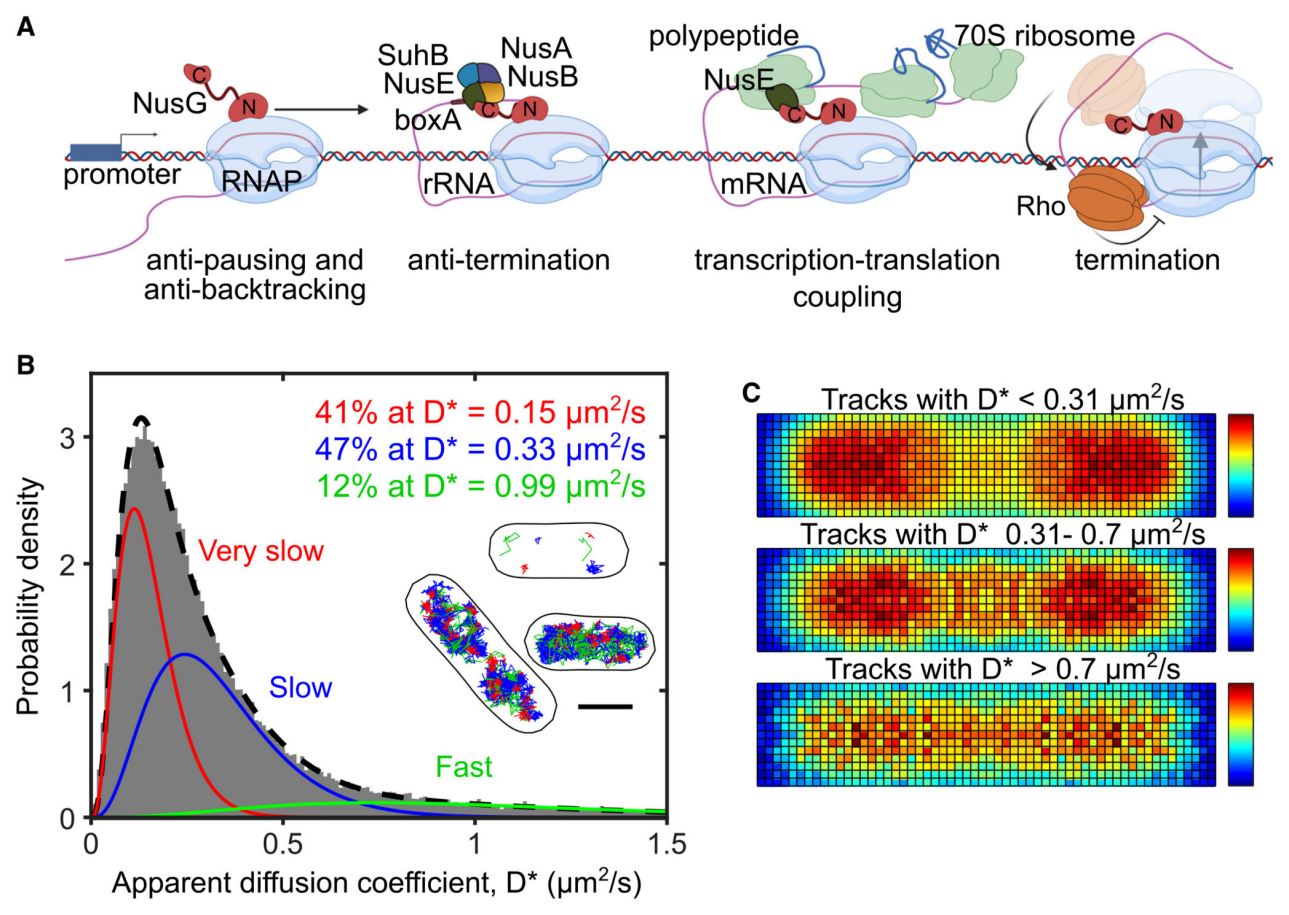
NusG main functions and diffusion landscape in living *E. coli* cells (A) Schematic representation of NusG functions and its main interactions during transcription elongation (see text for details). (B) Distribution of the apparent diffusion coefficients (D*) for 75,264 NusG molecules in live cells grown in M9GluVA. Average trajectory duration: 113 ms. The distribution is best fit by three populations with different mobilities: very-slow NusG (VS-NusG; in red) fixed at D*_vslow_ = 0.15 μm^2^/s; slowly diffusing NusG (S-NusG, in blue) fitted with a D*_slow_ = 0.33 μm^2^/s; and fast-diffusing NusG (F-NusG, in green) fitted with D*_fast_ = 0.99 μm^2^/s. Inset: representative examples of NusG tracks colored as red, blue, and green for VS-, S-, and F-NusG, respectively. For clarity, only 6 tracks are shown in the top cell. Scale bar, 1 μm. (C) Spatial distribution heatmaps of NusG tracks with a categorization threshold of D* = 0.31 for 400 cells with lengths of ~2–3 μm, having ~2 nucleoids. Top: a heatmap for molecules with D* < 0.31 μm^2^/s, representing mainly the VS-NusG species (accounting for 61% of all tracks). Middle: a heatmap for molecules with D* between 0.31 and 0.7 μm^2^/s, representing mainly the S-NusG species (83% of all tracks). Bottom: a heatmap for molecules with D* > 0.7 μm^2^/s, representing mainly the F-NusG species (87% of all tracks).

A key elongation factor, and the only one conserved throughout the tree of life, is bacterial protein NusG, with its archaeal and mammalian homolog being Spt4/5. In *E. coli*, NusG is a 21-kDa protein, which consists of two domains connected by a flexible linker; the NusG-NTD domain interacts with RNAP, whereas the NusG-CTD domain has many interacting and mutually exclusive partners.^4^ Functionally, NusG appears to play many roles in elongation (Figure 1A). NusG inhibits transcriptional pausing by enhancing transcription elongation processivity.^5^ NusG also forms an anti-termination complex (ATC) on ribosomal RNA (rRNA), which maintains steady rRNA transcription with the help of other proteins, including NusE, NusB, SuhB, and NusA.^6–9^ Further, NusG acts as a “bridge” to couple transcription with translation by linking RNAP to ribosomes via an interaction of NusE/S10 with the NusG-CTD.^10–13^ Finally, NusG stimulates termination factor Rho, an RNA helicase that terminates the synthesis of toxic gene products associated with homologous gene transfer and dormant phage genes embedded in the bacterial genome.^14,15^

Despite extensive study of the NusG roles in elongation, it is currently unknown how NusG distributes between all these activities in the cell, how the NusG distribution is affected by cell physiology, and whether NusG constitutes a limiting resource for the cell. This knowledge gap is further exacerbated by the fact that much of our understanding of NusG mechanisms is derived from *in vitro* work, therefore lacking the cellular context, the complexity of the chromosomal structure and organization, and the presence of competing processes. For example, there is controversy about the exact biological and mechanistic significance of recent cryoelectron microscopy (cryo-EM) structures of large transcription-translation coupled complexes, some of which contain NusG and some do not; the NusG presence also appears to depend on the length of nascent mRNA emerging from RNAP.^10,16^

Here, we advance our understanding of NusG functions by examining how NusG distributes between its various activities *in vivo*. To achieve this, we performed single-molecule tracking and super-resolution imaging of NusG fluorescent fusion proteins, an approach we previously used to study proteins involved in transcription, DNA repair, and chromosome organization.^17–21^ Under conditions supporting moderate growth rates, we found that most of NusG is present in a chromosome-associated population (via an elongating RNAP) and a slowly diffusing population corresponding to a NusG complex with free 30S ribosomal subunit; notably, there is little free NusG (F-NusG) in the cell. Using mutants of NusB and of NusE, we show that most chromosome-associated NusG is involved in anti-termination on rRNA genes, and in transcription-translation coupling. Finally, using a chemical treatment that removes clusters arising from liquid-liquid phase separation (LLPS), we show that NusG clusters are associated with LLPS-driven condensates involved in rRNA anti-termination.

## Results

### Construction and characterization of a functional PAmCherry-NusG fusion

To study the spatial distribution and mobility of NusG in bacterial cells, we first constructed a functional PAmCherry-NusG fusion in *E. coli* strain MG1655, where *nusG* is part of the essential *secE*-*nusG* operon. We first attempted to insert PAmCherry at the C terminus of NusG using λ red recombination,^22^ but no viable fusions were obtained. We then inserted a PAmCherry, along with a flexible linker, at the NusG N terminus; since this strategy required inserting the PAmCherry gene between *secE* and the *nusG* coding sequence (where having an antibiotic marker for successful integration was not possible), we used gene gorging.^23–25^ The resulting strain carrying PAmCherry-NusG exhibited normal growth compared with the wild type (WT) (Figure S1A); further, the fusion was expressed as an intact protein (Figure S1B).

### Single-molecule tracking reveals a wide range of NusG mobilities *in vivo*

To study NusG mobility and spatial distribution in live cells, we used single-molecule tracking combined with photoactivation localization microscopy (tracking PALM; Manley et al.^26^). Since NusG is a transcription elongation factor involved in many functions occurring on the bacterial chromosome, we expected NusG to distribute in at least two diffusive subpopulations; a very slowly moving species representing NusG molecules that bind stably with RNAP during transcription elongation on the chromosome (and therefore adopt the very low mobility of the chromosomal loci), and a fast-moving species representing F-NusG molecules (~50 kDa for the entire fusion construct). In our previous work on RNAP, we showed that tracking PALM is able to capture this entire range of intracellular mobilities.^17^

We first studied NusG mobility in M9 minimal media supplemented with glucose, MEM vitamins, and MEM amino acids (hereafter, “M9GluVA”). Using photoactivation and imaging using 10.64-ms exposures, we collected 75,264 single-molecule tracks, calculated their apparent diffusion coefficient (D*), and summarized the mobility of all tracks in a D* distribution (gray histogram, Figure 1B). The D* distribution showed that the large majority of molecules appear to have fairly low mobility (D* < 0.5 μm^2^/s). As with other DNA-binding proteins,^17,18,27^ the shape of the distribution is complex. We fitted our data to models containing different number of diffusing species and evaluated fits using residual analysis. The data could not be fit well by a single diffusing species (Figure S2A). A two-species free fit described the distribution better (Figure S2B), splitting approximately evenly between a very-slow species (D*_vslow_ ~ 0.15 μm^2^/s) and a slow species (D*_slow_ ~ 0.4 μm^2^/s).

To explore the possibility that our fitting above fails to capture a minor fast-diffusing species (with D* ~ 1 μm^2^/s), we also performed a three-species fit, with the D* of the very-slow species fixed to the value obtained by the two-population fit. The three-species fit fitted our distribution extremely well (Figures 1B and S2C) and showed, in addition to the two main species, the presence of a minor fast-diffusing species (D*_fast_ ~ 1.0 μm^2^/s) that accounts for ~12% of all NusG. Both two- and three-species fits showed that there is little fast-diffusing NusG in the cell, a species which we will refer to as F-NusG. We then tentatively assigned species with different mobilities to different activities or complexes of NusG. Since the very-slow species (~41% of all tracks; hereafter, the “VS-NusG” species) has a very low mobility, similarly to proteins that bind stably to the chromosome,^17^ we initially assigned this species to NusG molecules indirectly (and stably) associated with the chromosome via interactions with RNAP and, possibly, via other machinery that interacts with the chromosome during active elongation, anti-termination, or Rho-dependent termination.

Intriguingly, however, the largest fraction (~47%) of the tracks is due to the slow species (D* ~ 0.3 μm^2^/s; hereafter, the “S-NusG” species). This mobility is much slower than that expected for F-NusG, indicating that S-NusG comprises NusG molecules with slowed-down motion due to interactions with much larger structures. The structures are likely to be one or more of the three major interacting partners of NusG in elongation: the RNAP core, the Rho termination factor, and the ribosome or ribosomal subunits.

We also found that the distribution between the three main diffusive species in M9GluVA was similar to that in a medium supporting slower growth (M9 + 0.2% glucose medium, hereafter “M9Glu,” with a generation time of ~67 min; Figure S2D), as well as to a medium supporting faster growth (rich-defined medium, hereafter “RDM,” with a generation time of ~35 min).

To map the sub-cellular distributions of the NusG species and test our initial assignments, we divided the NusG tracks into three mobility bands: molecules with D* < 0.31 μm^2^/s (mainly VS-NusG), molecules with D* between 0.31 and 0.7 μm^2^/s (mainly S-NusG), and molecules with D* > 0.7 μm^2^/s (mainly F-NusG). We then plotted the spatial distributions as heatmaps (Figures 1C and S2E, for cell-size ranges featuring two nucleoids vs. one nucleoid, respectively). The VS-NusG distribution resembled the distribution of chromosome-engaged RNAPs, which tend to localize at the periphery of the nucleoid,^17^ and is consistent with the assignment of VS-NusG to NusG molecules engaged in transcription elongation.

In contrast, the S-NusG fraction is found throughout the cytoplasm and not exclusively in the nucleoid; F-NusG showed a similar trend. This localization pattern is different from the localization of diffusing RNAP, which we had previously shown to localize almost exclusively to the nucleoid region due to non-specific interactions with the chromosome.^17,28^ These results strongly suggest that the S-NusG is formed due to a NusG inter-action with a large structure other than RNAP. Further, our results are consistent with an interaction of NusG with the 30S free ribosomal subunit, since the latter localizes throughout the cell, and is not excluded from the nucleoid.^29^

### Characterizing the fast-diffusing NusG species

Our initial analysis showed that most NusG molecules interact with larger partners, leaving little F-NusG in the cell. To verify this observation, and determine more accurately the mobility of F-NusG, we overexpressed unlabeled NusG from an IPTG-inducible promoter (Mooney et al.^30^; STAR Methods) to out-compete PAmCherry-NusG from its interactions with its partners and release the fusion molecules in the cytoplasm.

**Figure 2:**
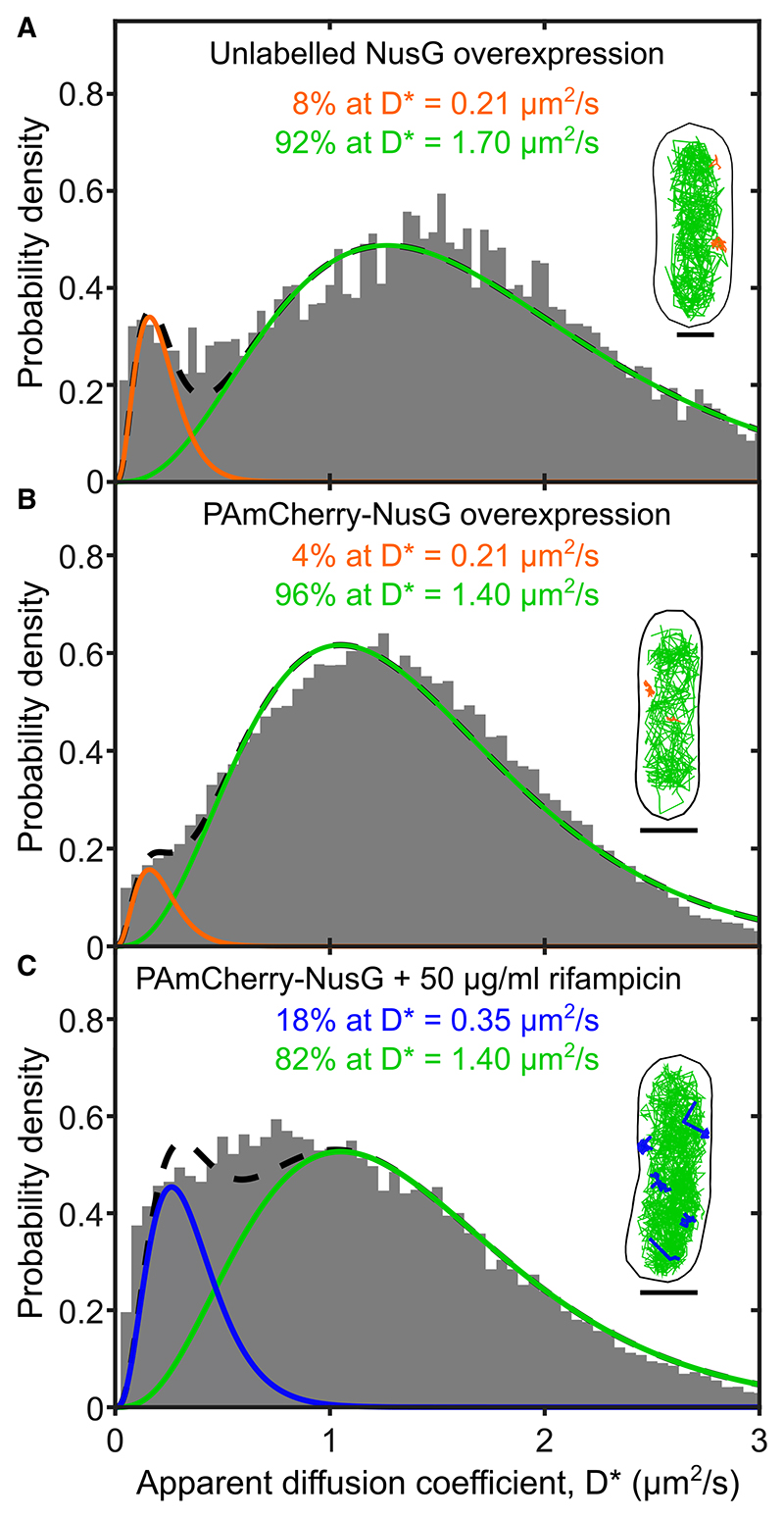
Measuring the intracellular mobility of fast-diffusing NusG in M9GluVA (A) D* distribution for 9,751 NusG molecules in live cells after overexpressing unlabeled NusG for 30 min post induction with 1 mM IPTG. Average trajectory: 84 ms. The distribution is best fit by two populations with D* of 0.21 and 1.7 μm^2^/s. Inset, representative examples of tracks corresponding to the two populations; the categorization threshold was 0.25 μm^2^/s. Scale bars, 1 μm. (B) D* distribution for 62,867 NusG molecules in live cells after overexpressing PAmCherry-NusG for 30 min post induction with 1 mM IPTG. Average trajectory: 84 ms. The fit and representative tracks were prepared as in (A). (C) D* distribution for 28,198 NusG molecules in live cells after inhibiting transcription initiation using 50 μg/mL rifampicin (Rif) for 30 min. Average trajectory: 86 ms. The fit and representative tracks were prepared as in (A), with the exception of the D* of the F-NusG being fixed at D*_fast_ =1.4 μm^2^/s, and the categorization threshold for track coloring being 0.35 μm^2^/s.

Indeed, upon overexpression of unlabeled NusG following 30 min induction, the NusG mobility distribution (Figure 2A) showed near complete disappearance of VS-NusG and S-NusG (<10% for the sum of the two species). Further, a fast-diffusing species (D*_fast_ ~ 1.7 μm^2^/s) became predominant, accounting for >90% of all NusG. These results showed that overexpressed NusG fully replaces the tagged version in its interactions in the cell and provides a better estimate (due to much better sampling than that in Figure 1B) of the mobility of F-NusG.

To further validate the mobility of F-NusG without relying on releasing NusG from its complexes, we also overexpressed a PAmCherry-NusG fusion from a low-copy-number plasmid with an IPTG-inducible *plac* promoter in WT MG1655. Western blots (Figure S3) showed that our conditions led to PAmCherry-NusG overexpression at levels ~5-fold higher that native NusG. After 30 min of induction, NusG appears almost exclusively (96%) as F-NusG (Figure 2B). This experiment also provided an additional estimate for F-NusG mobility (D* ~ 1.4 μm^2^/s).

To further support the analysis above and confirm whether VS-NusG indeed comprises NusG molecules bound to chromo-some-bound RNAP during transcription elongation, we treated cells with the antibiotic rifampicin (Rif) to block initial transcription and subsequent elongation^31,32^ and determined NusG mobility (Figure 2C; see also Figures S4A and S4B for fit evaluations). As with the NusG overexpression experiments, VS-NusG essentially disappears as a result of Rif treatment, showing that chromosomal association of NusG requires, as expected, entry of RNAP into transcription elongation.^17,25^ The Rif treatment also substantially increased the abundance of F-NusG (from 12% to 85% of the entire NusG pool), as was seen after NusG overexpression (Figures 2A and 2B). Notably, the S-NusG species was reduced but not abolished by Rif treatment (still accounting for ~18% of all NusG), showing that its presence is not dependent on active transcription.

### Most chromosome-associated NusG is engaged in rRNA anti-termination

**Figure 3:**
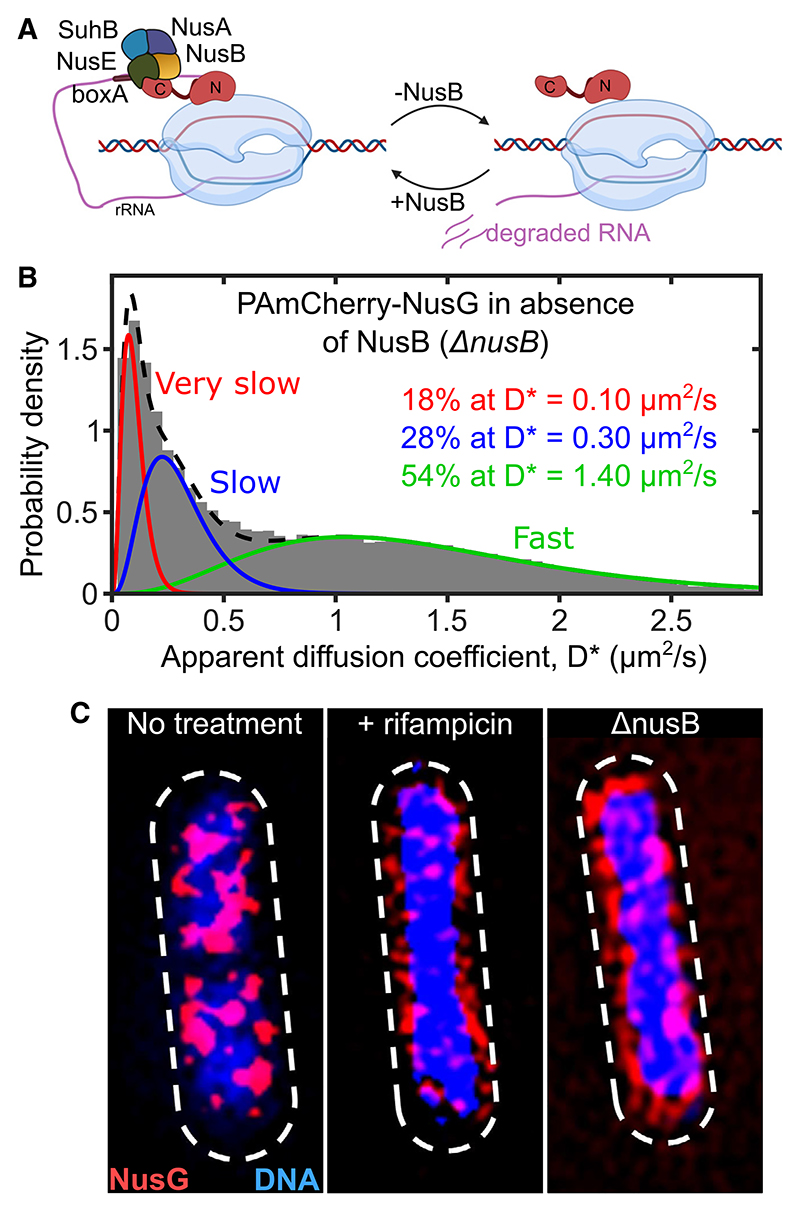
Blocking anti-termination complex formation increases NusG diffusion and alters its spatial distribution (A) Schematic depicting the effects of *nusB* deletion (Δ*nusB*) on *rrn* anti-termination. NusB deletion blocks anti-termination complex formation, depletes NusG from *rrn* operons, and leads to premature transcription termination and increased RNA degradation. (B) D* distribution for 41,524 NusG molecules in live cells deficient in anti-termination complex formation due to *nusB* deletion; the growth medium is M9GluVA. Average trajectory: 73 ms. The distribution is best fit by three populations with D*_vslow_ = 0.1 μm^2^/s (in red), D*_slow_ = 0.3 μm^2^/s (in blue), and D*_fast_ = 1.4 μm^2^/s (in green). (C) 3D-SIM imaging of sfGFP-NusG (in red) and DNA stained with DAPI (in blue) for untreated live cells, Rif-treated cells, and Δ*nusB* cells, showing the relative NusG spatial distribution in relation to the nucleoid, and highlighting how both Rif treatment and NusB deletion affect the NusG distribution and nucleoid decompaction. Dashed line: cell outlines.

We then examined what fraction of NusG engages in transcription anti-termination. Processive anti-termination is mediated by a complex of proteins interacting with RNAP to counteract premature termination^7,33^ and ensure efficient *rrn* operon transcription. This is accomplished by the formation of an *rrn* ATC, which includes NusA, NusB, NusE, NusG, and SuhB. ATC formation is initiated by the NusB:NusE heterodimer binding to a *boxA* site at the leader rRNA sequence (Figure 3A; see also Huang et al.,^6^ Huang et al.,^7^ Krupp et al.,^8^ and Burmann et al.^34^). NusB is found at copy numbers that are 50%–80% of those of core RNAP in the cell.^35^ If NusB is deleted, the assembly of ATC on *rrn* operons is blocked, reducing the total RNA content in the cell and increasing Rho-dependent termination.^36^ We thus reasoned that, by eliminating NusB, we can prevent anti-termination and use the ensuing change in NusG mobility to estimate the fraction of NusG involved in anti-termination.

To study NusG diffusion in the absence of NusB, we studied the NusG mobility distribution in a D*nusB* strain (Figure 3B). NusB deletion resulted in a large increase in F-NusG (which reached 54% of all NusG), in large part due to a ~60% decrease in VS-NusG (from 41% to 17%). This fraction (60%) serves as a lower bound of the fraction of chromosome-associated NusG that is involved in *rrn* anti-termination, since some VS-NusG freed from anti-termination may be directed to other (non-*rrn*) chromosome-associated species. Notably, the S-NusG species remains present, albeit reduced by ~40%. This reduction is consistent with our assignment of S-NusG to a NusG-30S complex, since loss of rRNA transcription will also reduce the levels of the mature 30S ribosomal subunit (due to large defects in 23S synthesis, which deplete 50S and 70S ribosomes^37^ and, in turn, deplete ribosomal proteins), and the levels of the proposed NusG-30S complex. However, these results do not exclude the presence of a putative NusG-Rho complex in the S-NusG species.

We also examined the spatial distribution of the NusG fraction involved in anti-termination using 3D structured illumination microscopy (3D-SIM; see STAR Methods). Using a sfGFP-NusG strain grown in M9GluVA, we observed that NusG localized in large clusters (10–20 clusters per cell) that decorate an irregular nucleoid (blue density; Figure 3C, left). This distribution was similar to that of RNAP in rich media, as we had previously observed using SIM.^17^ In contrast, Rif treatment removed all large NusG clusters, leaving mainly small regions of nucleoid-peripheral NusG signal over a decondensed nucleoid (Figure 3C, middle).

The *nusB* deletion also resulted in loss of most large NusG clusters, decondensed the nucleoid, and led NusG to a more regular nucleoid-peripheral localization (Figure 3C, right). The change in the NusG spatial distribution is consistent with the expectation that absence of *rrn* anti-termination will lead to a nucleoid-wide loss of the high levels of transcription of *rrn* operons and increased Rho termination. The remaining clusters are likely to represent NusG attached to clusters of mRNA-transcribing RNAPs, which should be less affected by the loss of *rrn* anti-termination.

### Loss of NusG contacts with the 30S ribosome releases NusG from elongation complexes and the S-species

We then examined the involvement of NusG in transcription-translation coupling, a process extensively studied *in vitro* via biochemical assays and structural approaches.^10,12,16,38^

**Figure 4:**
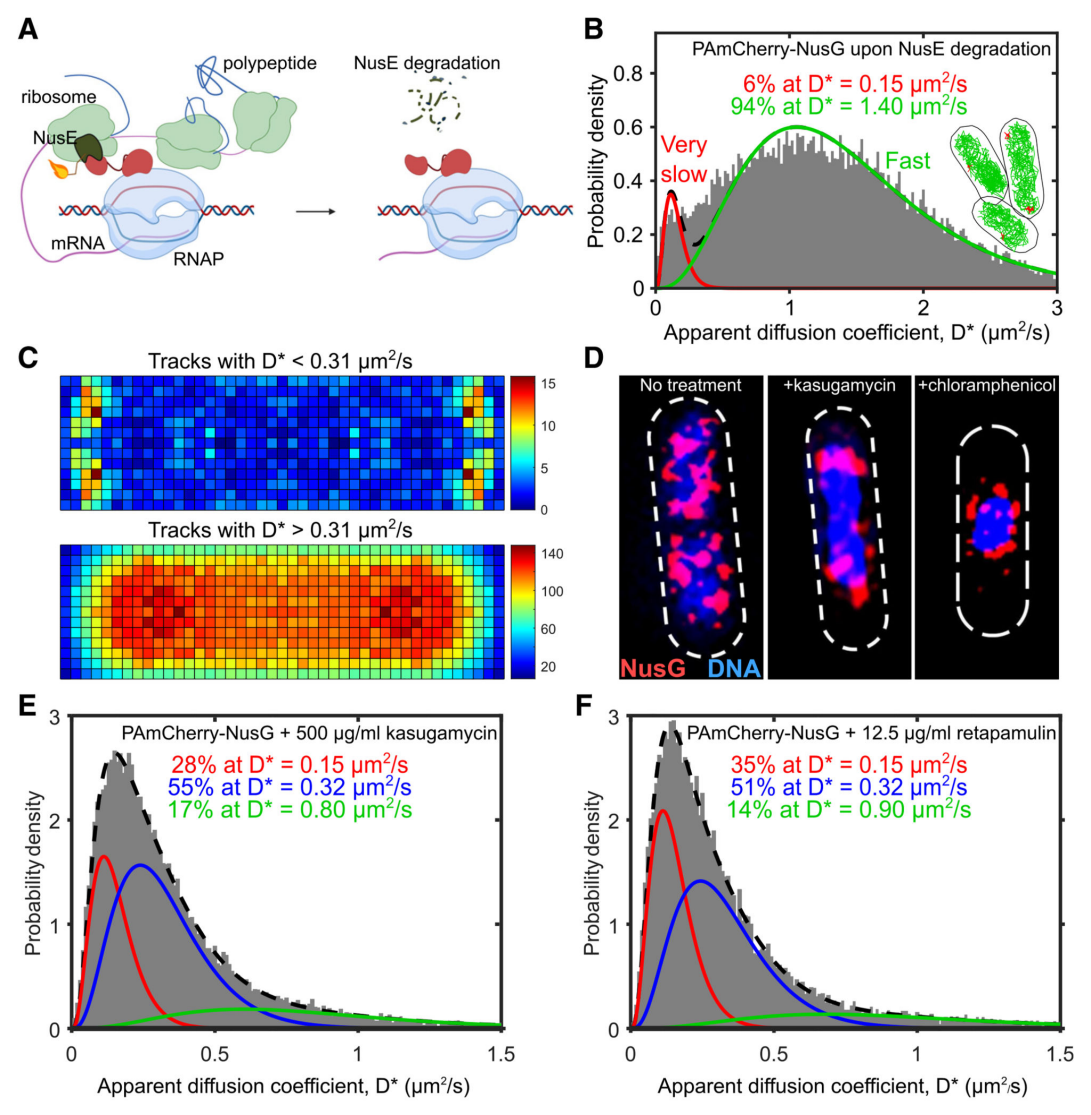
Effects of perturbing ribosome-NusG and ribosome-RNA interactions on the NusG spatial and mobility profile (A) Schematic of interactions between a lead ribosome and NusG in the context of transcription elongation; the interactions are between the ribosomal subunit NusE/S10 and the C-terminal domain of NusG. The fuse depicts the degron tag that leads to NusE degradation (depicted as dashed line in the schematic on the left) upon IPTG addition. (B) D* distribution for 20,593 NusG molecules in live cells after induction of NusE degradation (1 h in liquid media, as well as during imaging); the growth medium is M9GluVA. Average trajectory: 89 ms. The distribution is best fit by two populations with D*_vslow_ = 0.15 μm^2^/s (in red), and D*_fast_ = 1.4 μm^2^/s (in green). Inset, representative examples of tracks corresponding to the two populations (categorization threshold of D* = 0.31). (C) Spatial distribution heatmaps of NusG tracks with a categorization threshold of D* = 0.31 for 311 cells with lengths of ~2–3 μm, having ~2 nucleoids. Top: a heatmap for molecules with D* < 0.31 μm^2^/s, representing mainly the VS-NusG species (82% of all tracks). Bottom: a heatmap for molecules with D* > 0.31 μm^2^/s, representing the F-NusG species (99% of all tracks). () 3D-SIM imaging of sfGFP-NusG (in red) and DNA stained with DAPI (in blue) for untreated live cells, cells treated with 500 μg/mL translation-initiation inhibitor kasugamycin, and 100 μg/mL translation-elongation inhibitor chloramphenicol. Dashed line: cell outlines. (E) D* distribution for 31,592 NusG molecules in live cells after 30 min exposure to 500 μg/mL kasugamycin. Average trajectory: 121 ms. The distribution is fit by three populations with D*_vslow_ ~ 0.15 μm^2^/s (in red), D*_slow_ ~ 0.32 μm^2^/s (in blue), andD*_fast_ ~ 0.8 μm^2^/s (in green). (F) D* distribution for 18,000 NusG molecules in live cells after 30 min exposure to 12.5 μg/mL retapamulin. Average trajectory: 121 ms. The distribution is fit by three populations with D*_vslow_ ~ 0.15 μm^2^/s (in red), D*_slow_ ~ 0.32 μm^2^/s (in blue), and D*_fast_ ~ 0.9 μm^2^/s (in green).

To dissect the role of NusG-ribosome interactions mediated via the NusE/S10 subunit on the 30S ribosome (Figure 4A), we inserted a degron tag in the NusE C terminus (see STAR Methods). NusE interacts with NusG both during transcription-translation coupling, and during the assembly of the *rrn* ATC. As a result, once NusE is degraded, we expected a large increase in F-NusG; further, if indeed NusE interacts with NusG in the context of the putative NusG-30S complex, we expected S-NusG species to decrease substantially upon NusE degradation.

Indeed, upon induction of NusE degradation, the sum of VS-NusG and S-NusG populations was reduced from ~90% to less than 10%, with the large majority of NusG converted to F-NusG (D* ~ 1.4 μm^2^/s; Figures 4B, S4C, and S4D). These results strongly support our proposal that the S-NusG species corresponds mainly to a NusG-30S complex, and not to a NusG-Rho complex or to a NusG-RNAP, both of which should have been either unaffected or increased upon NusE degradation.

To explore further the effects of NusE degradation on NusG functions, we also examined the NusG spatial distribution after NusE degradation (Figure 4C) and established that the F-NusG population distributes across the cytoplasm (Figure 4C, bottom). In contrast, the few remaining low-mobility NusG molecules are found in the nucleoid periphery (Figures 4C, top and S5) and likely result from RNAs prematurely aborted by Rho, and from any remaining NusG-RNAP complexes.

We then used 3D-SIM to determine the NusG sub-cellular distribution after destabilizing the NusG interactions with RNAP and ribosomes by blocking different steps in translation, while not affecting *rrn* anti-termination directly. We reasoned that if NusG couples RNAP with the leading ribosome, blocking translation would remove NusG-ribosome interactions and thus decrease the overall affinity of NusG for the elongation complex on mRNA genes. Previous work on the spatial distribution of ribosomes relative to the nucleoid had shown that treatment with chloramphenicol (Cam), a translation-elongation inhibitor, leads to filling of most cytoplasmic space with ribosomes, with the nucleoid becoming condensed into a spherical object appearing at mid-cell.^39^ Conversely, kasugamycin (Kas), a translation-initiation inhibitor, inhibits 70S ribosome assembly while allowing the nucleoid to maintain most of its length.^39^

After Kas treatment, we observed that medium-size NusG clusters, previously distributed peripherally and along the nucleoid, were lost (cf. Figure 4D, left with Figure 4D, middle). In contrast, the large NusG clusters located at the nucleoid edges closest to the cell poles remained unaffected and are likely to reflect NusG bound to *rrn* operons. On the other hand, Cam treatment led to a spherical nucleoid surrounded by NusG clusters (Figure 4D, right); this NusG localization resembled closely the RNAP distribution seen in cells treated with Cam.^17,28^

To explore further the effects of translation-initiation inhibition (which should affect 70S formation), we examined the effect of two translation-targeting antibiotics, Kas and retapamulin (Ret) on the NusG D* distribution (Figures 4E, 4F, and S6). Kas and Ret both decrease VS-NusG compared with the unperturbed cells, but to a different degree (by ~32% and ~15%, respectively; cf. with Figure 1B); the VS-NusG decrease is likely due to the loss of NusG involved in transcription-translation coupling. The difference between Kas and Ret treatments may reflect their different modes of action; Kas acts on the 30S subunit, preventing translation initiation by causing dissociation of fMet-tRNA and destabilization of the 30S-mRNA complex or by blocking the 70S complex from leaving the start codon,^40,41^ whereas Ret prevents formation of active 50S subunits (and does not destabilize the initial 30S-mRNA complex). In contrast, the S-NusG species appears unaffected, likely reflecting the stability of the 30S ribosome over the course of the Kas and Ret treatments.

### A large fraction of NusG is confined in biomolecular condensates that may form via liquid-liquid phase separation

Our NusG diffusion analysis showed that deletion of *nusB* (Figure 3B) and consequent failure to assemble *rrn* ATCs resulted in a large decrease (~60%) in the abundance of VS-NusG. This decrease was also accompanied by an apparent decrease in the mobility of the remaining VS-NusG from a D* ~ 0.15 to ~0.10 μm^2^/s (Figure 3B).

To understand the origin of this decreased mobility, we considered the findings of Ladouceur and co-workers,^42^ where *nusB* deletion led to loss of large RNAP clusters *in vivo*. The same work showed that anti-termination factor NusA, which can form phase-separated liquid droplets *in vitro*, can drive foci formation *in vivo*, and may drive LLPS of RNAPs and components of the *rrn* ATC *in vivo*. To test for the presence of condensates, Ladouceur et al. treated cells with 1,6-hexanediol (HEX), an aliphatic alcohol that destabilizes liquid condensates but not protein aggregates.^43^ HEX exposure induced loss of RNAP and NusA clustering, providing strong evidence for RNAP/ NusA LLPS *in vivo*.

**Figure 5:**
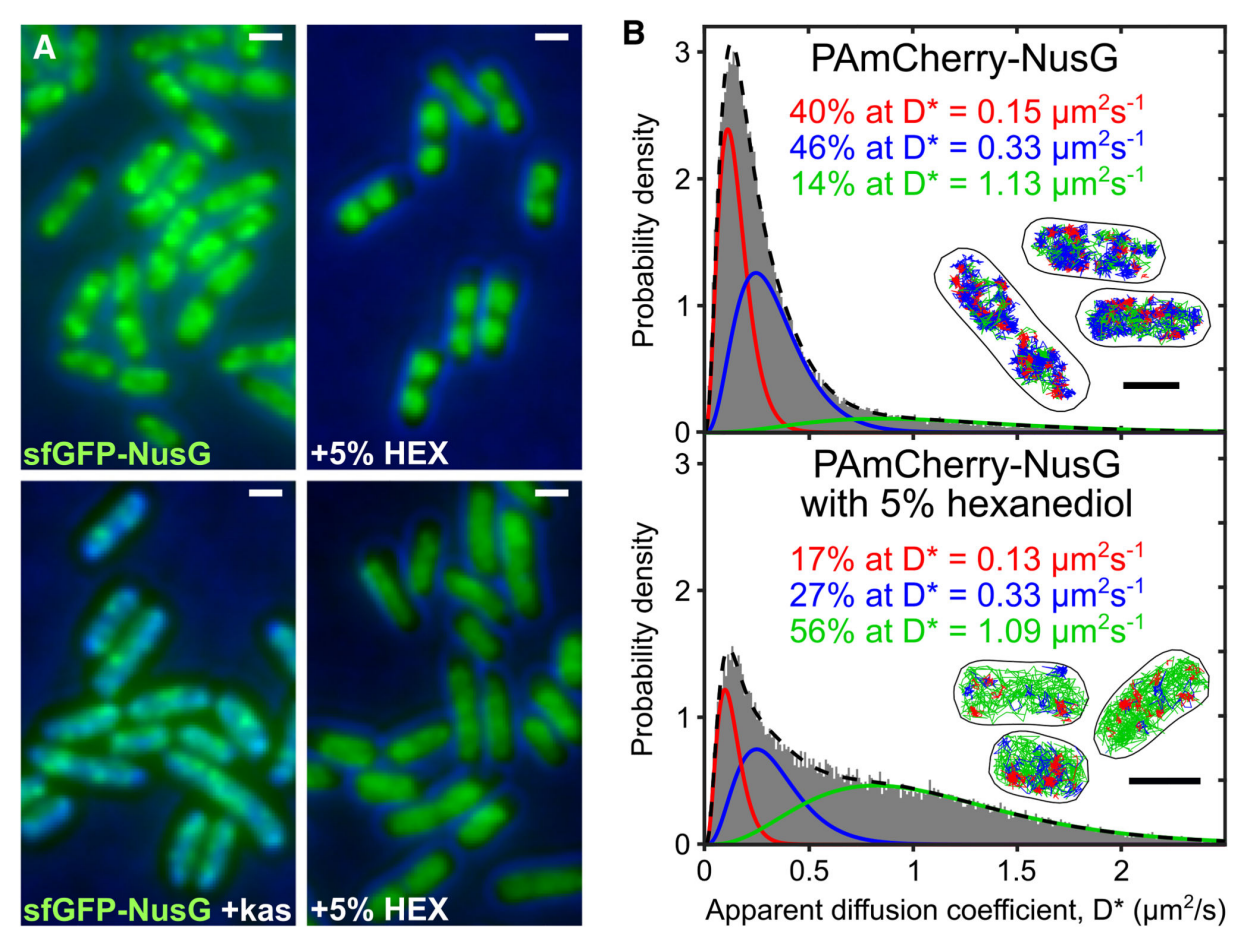
Treatment with 1,6-hexanediol eliminates NusG clusters and releases NusG from biomolecular condensates involved in transcription anti-termination (A) sfGFP-NusG images of live cells imaged using 10-ms exposures. Results obtained from cells grown in M9GluVA media until OD600 ~ 0.2 and split into cells that remain untreated and cells that were treated for 30 min in liquid culture with kasugamycin. Both untreated and Kas-treated cells were then immobilized on agarose pads with or without 5% 1,6-hexanediol (HEX) for 5 min prior to imaging. (B) D* distribution for live cells containing PAmCherry-NusG in the absence (top) and presence of 5% HEX for 5 min (bottom) for 75,264 and 36,241 NusG molecules, respectively. Average trajectory: 103 ms. The D* for untreated cells is similar to that in Figure 1B; the D* distribution for HEX-treated cells is best fit by three populations with D*_vslow_ = 0.13 μm^2^/s (in red), D*_slow_ = 0.33 μm^2^/s (fixed; in blue), and D*_fast_ = 1.09 μm^2^/s (in green). Inset, representative examples of tracks corresponding to the three populations; the categorization thresholds were D* < 0.2 μm^2^/s for the red tracks, 0.2 < D* < 0.5 μm^2^/s for the blue tracks, and D* > 0.5 μm^2^/s for the green tracks. Scale bars, 1 μm.

To test whether NusG clustering is linked to RNAP/NusA condensates, we grew cells expressing sfGFP-NusG in M9GluVA. Cells were then immobilized on agarose pads with and without 5% HEX, incubated for 5 min, and immediately imaged (Figure 5A, top panels). Untreated cells showed large NusG clusters in the form of bright spots within the cytoplasm, whereas HEX-treated cells sfGFP-NusG clusters disappeared completely, leading to the loss of NusG clustering. We repeated the experiment with cells pre-treated with Kas in liquid culture for 30 min, a treatment that is expected to retain mainly the NusG clusters involved in *rrn* ATCs (Figure 4D) before immobilization on agarose. As predicted, Kas-treated cells displayed the NusG clusters more clearly (Figure 5A, bottom left), while subsequent HEX treatment greatly diminished NusG clustering. These results strongly suggest that the NusG clusters formed are part of biomolecular condensates.

To further test whether the NusG clusters belong to condensates sensitive to environmental changes (in contrast to being simply part of stable aggregates), we tested whether NusG condensate dissolution is rapidly reversible. We thus compared the spatial distributions of mNeonGreen-NusG (MNG-NusG) in HEX-treated cells, and in cells where HEX was subsequently washed away (Figure S7A). Within 5 min after removing HEX, condensates fully recovered, demonstrating the dynamic nature of NusG-containing condensates; a similar pattern was seen for RNAP.^42^ These results strongly support that there is a substantial presence of NusG in LLPS-driven condensates of the *rrn* anti-termination machinery *in vivo*.

We also examined MNG-NusG cells for evidence of NusG cluster splitting and fusion. Although such behaviors are characteristic of many LLPS condensates, we did not anticipate many fusion events, since the clusters are anchored to the chromosome (on *rrn* operons) and show limited mobility (akin to that for RNAP^17^). To test this prediction, we performed time-lapse imaging (1-s images every 5 s for ~1 min, with the timescale limited by photobleaching; Video S1). Most clusters indeed show limited mobility; further, we observed only few events of cluster splitting and fusion (Figure S7B).

To quantify the effect of HEX treatment on NusG condensates, we examined the NusG mobility by performing tracking PALM on HEX-treated cells expressing PAmCherry-NusG in M9GluVA (Figure 5B). Treatment by HEX dramatically changed the NusG diffusion profile. First, the abundance of VS-NusG decreased by ~60%, from 41% in untreated cells to 17% in HEX-treated cells. Second, the abundance of F-NusG species increased dramatically, from 12% in untreated cells to 56% in HEX-treated cells. Third, the abundance of S-NusG also decreased by ~40%.

This striking decrease of VS-NusG in the HEX-treated cells suggests that, under moderate growth conditions, ~25% of all NusG is confined in condensates. The results were also consistent with the Δ*nusB* results (Figure 3B), which showed an identical decrease in the abundance of VS-NusG and S-NusG species, as well as a similar reduction in the mobility of the VS-NusG. Taken together, the results of the HEX treatment and the *nusB* deletion clearly establish that these conditions disrupt the condensates in a similar fashion, and free the condensate-confined pool of NusG.

## Discussion

Here, we combine our powerful single-molecule tracking approach with mutational analysis *in vivo* to map the spatial distribution and dissect the functions of transcription elongation factor NusG using *E. coli* as a model organism. Our analysis complements the extensive *in vitro* analysis of NusG activities and interactions using structural, biochemical, and biophysical approaches, as well as *in vivo* analysis using genetic and cell-biology approaches. Our results offer direct views of the allocation of the NusG pool between its many functions and provide estimates that can help model transcription and gene expression in bacterial cells. Since NusG is the only transcription factor conserved among all kingdoms of life, many of our conclusions are likely to hold true for many organisms other than *E. coli*. Our approach also provides a roadmap on how to analyze activities of multi-functional proteins *in vivo* using imaging.

### Only ~10% of NusG is free in the cell

NusG is a multi-functional global elongation factor involved in many complexes nucleated on RNAP molecules during transcription elongation. It was unknown, however, what fraction of NusG is involved in such chromosome-associated complexes under different growth conditions. Our work shows that for media supporting doubling times of 35–67 min, there is a limited amount of F-NusG (corresponding to the F-NusG species) in the bacterial cytoplasm, with only ~10% of NusG appearing to diffuse rapidly. This result strongly suggests that NusG is a limited resource for the cell and predicts the presence of a dynamic competition between machineries for binding NusG, with the split between activities changing according to the cellular requirements for growth, duplication, and adaptation. Notably, if NusG binds to partners such as NusE and diffuses as a heterodimer, the resulting complex (~60 kDa) would still be classified as part of F-NusG.

### F-NusG binds to the chromosome non-specifically and transiently for >50% of time

Our work clearly established that free PAmCherry-NusG, a protein of ~48 kDa, has an apparent diffusion coefficient of D*_fast_ ~ 1.4 μm ^2^/s (Figure 2). Given that NusG is not known to bind directly to chromosomal DNA but instead binds to the chromosome indirectly (via RNAP during transcription elongation), we would expect that the D* value and diffusion behavior of NusG should be independent of the presence of the chromosome, as we have shown for proteins lacking DNA-binding domains.^20^ However, the D* value for F-NusG matches that for a 4-times larger protein that is unable to bind DNA (Lac^-41^, a ~200 kDa truncated *lac* repressor (LacI) derivative lacking its DNA-binding domain).

Further, under the same consideration that NusG does not bind directly to chromosomal DNA, the diffusion of F-NusG in cells with intact nucleoids should resemble the behavior of HU-PAmCherry, 48 kDa (same size as PAmCherry-NusG) in cells where the chromosomal DNA has been degraded (DNA-free cells; see Stracy et al.^20^). Again, strikingly, while the estimated accurate diffusion coefficient of HU-PAmCherry in DNA-free cells is D_acc_ ~12.6 μm^2^/s,^20^ the D_acc_ for PAmCherry-NusG in cells with intact nucleoids is estimated to be ~3.5 μm^2^/s (based on the similarity of the D* values for PAmCherry-NusG and LacI-PAmCherry, and the conversion of D* to D_acc_ for LacI-PAmCherry using simulations of diffusion^20^).

These results strongly suggest that either NusG interacts in the cell with another diffusing biomolecule such as a high-copy-number protein, e.g., NusA, shown to interact *in vitro* with NusG,^44^ or that NusG binds non-specifically and transiently to chromosomal DNA. The strong nucleoid-like localization of F-NusG (Figures 2B and 2C) supports the second hypothesis. Further, the fact that the F-NusG species persists and shows no change in mobility in Rif-treated cells, where RNA is highly depleted, indicates that these non-specific interactions are not mediated primarily by nascent RNA. As a result, we can use the estimated D_acc_ for NusG in the absence and presence of the nucleoid (~12.6 and ~3.5 μm^2^/s, respectively) to estimate that F-NusG binds to the chromosome transiently for ~70% of its diffusion time (STAR Methods). We note that interactions between the DNA and NusG-like proteins, especially with non-template DNA in the context of the transcription bubble, have been previously discussed.^45^ The non-specific NusG interactions with the chromosome may increase the effective NusG concentration in the vicinity of elongation complexes, thus facilitating the search of NusG for some of its targets.

### NusG interacts with the 30S ribosomal subunit before translation initiation

Our work establishes that most NusG associates with transcription elongation complexes on the chromosome (forming VS-NusG species), or diffuses as part of larger complexes (i.e., S-NusG species). Notably, NusG binds to the nucleoid stably only in the presence of transcription elongation, as shown by Rif treatment (Figure 2C); this is further supported by the similarity of the spatial distributions of VS-NusG (Figure 1C, top) with chromosome-associated RNAP.^17^

Our detection of an abundant S-NusG species is intriguing. Our NusE degradation results (Figure 4) ruled out free RNAP and Rho as large protein partners of NusG in S-NusG. We also rule out the fully assembled 70S ribosome as the potential partner, since the slow-diffusing species has full access to the nucleoid, in contrast to the 70S ribosome, which does not enter the nucleoid in a diffusing form.^29^

Instead, the only large complex that can access the nucleoid and interact with NusG is the 30S ribosomal subunit, which is not excluded from the nucleoid and has a spatial profile and diffusion coefficient similar to that we observe here (D* ~0.3– 0.4 μm^2^/s for 30S^28,29,39^). Our NusE degradation results further support the presence of NusG-30S complex, since loss of NusE essentially eliminates S-NusG. Consistent with this interpretation, structural studies suggest that NusG:NusE interaction occurs both in the context of the 70S ribosome, and in the context of free 30S.^38^ Our assignment of S-NusG to a NusG-30S complex is also supported by our results showing that S-NusG levels are unaffected by translation inhibition (Figures 4E and 4F).

The presence of substantial amounts of a NusG-30S complex also suggests an additional major route of NusG entry to the transcription elongation complex—that of association during translation initiation through the interaction of a NusG-loaded 30S with the Shine-Dalgarno sequence on mRNA, formation of 70S ribosomes, ribosome translocation toward an elongating (or stalled) RNAP, and NusG-facilitated transcription-translation coupling on target mRNA genes. This is in line with the chromatin immunoprecipitation (ChIP)-chip analysis^25^ showing that NusG enters the transcription elongation complex on mRNA a few hundred bp from the transcription start site.^25^ This 30S-guided mode of entry is in addition to a simpler mode where NusG enters transcription elongation by F-NusG binding directly to RNAP molecules after they enter elongation, with an *in vitro* measured K_D_ of ~120 nM^46^).

### Most NusG is involved in transcription anti-termination on rRNA genes and in transcription-translation coupling

NusG is known to interact with NusB, NusE, and other proteins to form an ATC on *boxA* sequences at the 5^0^ end of rRNA, and in turn, protect rRNA from premature termination.^15,33^ Since NusB:NusE dimerization on *boxA* is the pre-requisite for ATC assembly, *nusB* deletion results in 95%–97% reduction in anti-termination in rRNA genes, the expression of which accounts for the majority of RNA in cells growing at moderate growth rates.^47^ Intriguingly, upon *nusB* deletion, the NusG copy number increases, suggesting that, in the absence of ATC, NusG levels elevate so it can act as processivity elongation factor and salvage some level of *rrn* transcription.^37^

Our mobility analysis showed that *nusB* deletion leads to a 60% reduction of NusG engaged with the elongation complex, which we attribute to loss of the ATC on rRNA operons, subsequent termination, and loss of NusG as elongation complexes dissociate after being prematurely terminated by Rho.^9,33,37,48^ This interpretation is further supported by our super-resolution analysis (Figure 3C), which showed that *nusB* deletion leads to a general decompaction of the nucleoid and a loss of the large NusG clusters located in the nucleoid periphery, where the *rrn* operons are expected to reside.^49,50^ We have also visualized these *rrn* anti-termination NusG clusters directly by halting translation initiation with Kas. Our results establish that, at moderate growth rates, most NusG molecules on the chromosome are involved in translation-independent activities, and specifically in rRNA anti-termination. This NusG fraction engaged in *rrn* anti-termination is expected to increase further in cells grown in richer media, such as LB and RDM.

The reduction of NusG engagement with the elongation complex is even more dramatic (~90%) when both *rrn* anti-termination and transcription-translation coupling are eliminated by removing the NusG-NusE interactions (using the NusE degron). This comparison strongly suggests that the involvement of NusG in transcription-translation coupling *in vivo* is substantial, with coupling occupying the second largest fraction of the NusG pool after *rrn* anti-termination.

### NusG molecules involved in *rrn* anti-termination reside in phase-separated condensates

The *nusB* deletion (Figure 3B) reduced the bound NusG fraction, and, intriguingly, yielded an even slower VS-NusG population at ~0.1 μm ^2^/s. This striking result suggested that the bound population in unperturbed cells was a convolution between a DNA-bound state, and a state that had some limited mobility and exhibited confined diffusion, as was shown for RNAP and NusA in the large clusters forming during exponential growth in rich media.^17,42^ This same work proposed that RNAP clusters involved in *rrn* anti-termination are biomolecular condensates formed via LLPS, and that NusA, a protein with disordered segments, may nucleate condensate formation *in vivo*.

Our observations agree with the Ladouceur et al. work and extend its findings. We show that a HEX treatment was able to dissolve NusG clusters both in Kas-treated cells (enriched for rRNA transcription and devoid of mRNA transcription), as well as in untreated cells (Figure 5A); the clusters, as for RNAP, are dynamic and recover quickly when HEX is removed. Our results show directly that NusG is part of *rrn* anti-termination condensates in *E. coli*, which we also expect to include NusA and other anti-termination factors. Further, our tracking analysis showed that HEX treatment yielded an almost identical diffusive profile to the one obtained with *nusB* deletion in terms of loss of VS-NusG species and correlated increase of F-NusG species (compare Figures 3A and 5B), strongly suggesting that *rrn* synthesis occurs almost exclusively in LLPS condensates, providing further strong links between condensate formation and *rrn* anti-termination.

### Model of NusG functional allocation *in vivo*

**Figure 6:**
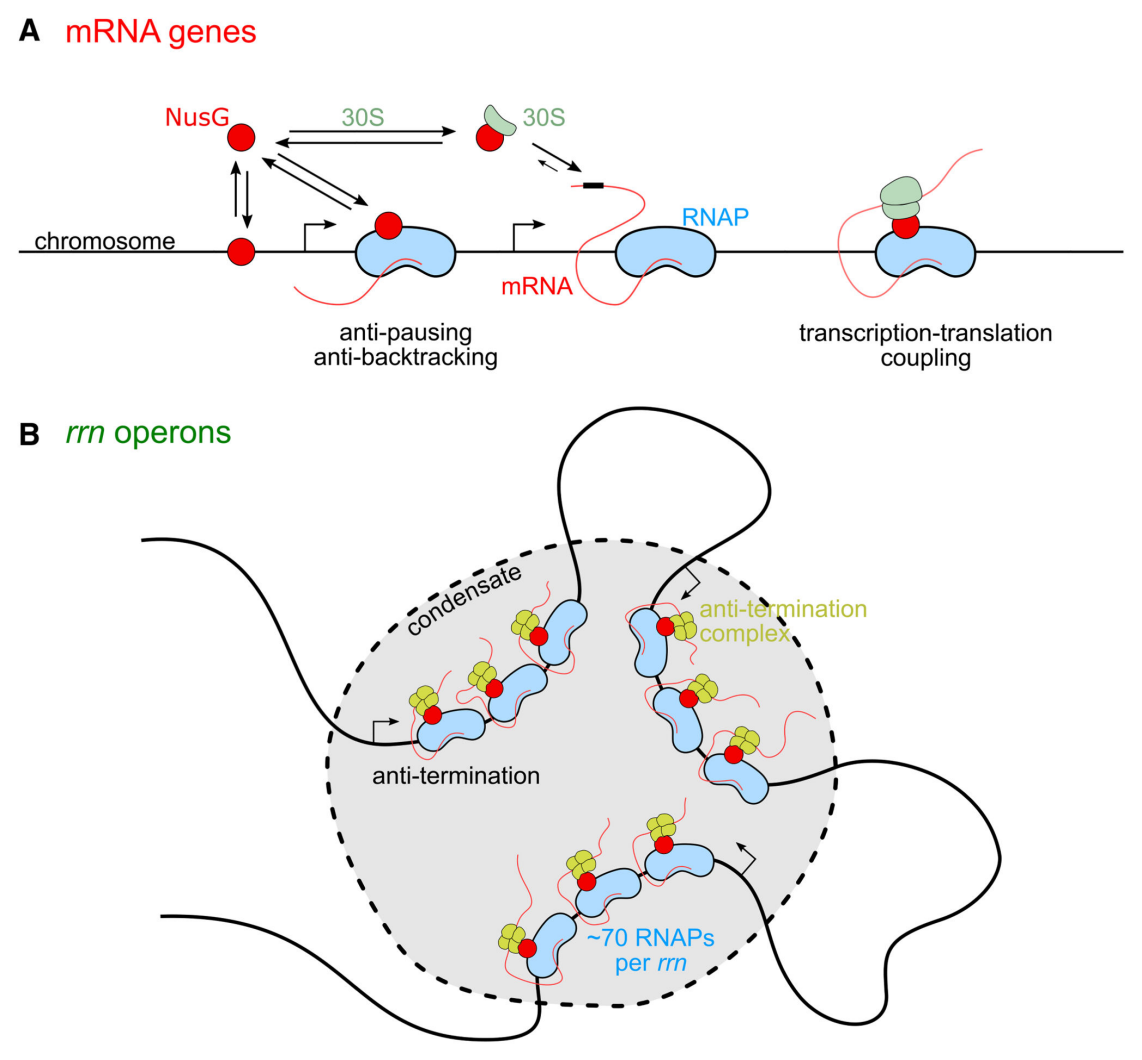
Model of NusG functional distribution inside bacterial cells (A) Functional distribution of NusG on mRNA genes. Before locating its target (a transcription elongation complex), NusG interacts non-specifically and transiently with the bacterial chromosome. NusG can enter the elongation complex by binding to RNAP directly. NusG also forms an abundant complex with free 30S ribosomal sub-unit, which can interact with the mRNA during translation initiation and offer a route for locating the elongating (or paused) RNAP and establishing transcription-translation coupling. (B) Role of NusG in rRNA transcription. *rrn* operons are heavily transcribed in moderate-to-fast growth rates, with each *rrn* operon being occupied by tens of RNAP molecules (~70 for exponential growth in rich media). NusG forms part of the *rrn* anti-termination complex that ensures fast and complete synthesis of rRNA. The *rrn* operons are in close proximity in 3D space and form part of anti-termination transcriptional condensates forming via LLPS. Most NusG molecules during moderate-to-fast growth rates are occupied in these large transcriptional assemblies.

Our results provide a working model for how NusG distributes between its functions in cells to modulate transcription in *E. coli* (Figure 6). NusG in the cell associates with larger structures and machinery and is a limiting for NusG-dependent reactions. This landscape creates opportunities for functional modulation of different genes by regulation of the concentration of NusG complexes (on the chromosome and in the cytoplasm) in different physiological states.

On mRNA genes (Figure 6A), NusG can enter the elongation complex by binding to RNAP directly. NusG also forms an abundant complex with free ribosomal subunit 30S, which can interact with the mRNA during translation initiation and offer a route for locating elongating or paused RNAPs, and establishing transcription-translation coupling. Disruption of the NusG-ribosome interaction affects NusG-dependent coupling and leads to loss of NusG from the transcription elongation complex, which may lead to transcription termination. NusG molecules bound to RNAP during mRNA transcription will also interact transiently with Rho when Rho-dependent termination occurs, e.g., at the ends of some genes.

On rRNA genes (*rrn* operons; Figure 6B), which are heavily transcribed during moderate-to-fast growth, NusG forms part of the *rrn* ATC that ensures that all RNAPs achieve rapid and complete synthesis of rRNA. The *rrn* operons are in close proximity in 3D-space and form part of anti-termination transcriptional condensates generated via LLPS. Most NusG molecules under moderate-to-fast growth rates are occupied in these large transcriptional assemblies. Our data also suggest that a large fraction of NusG molecules in the condensates displays confined diffusion within the condensates (as also shown for RNAP) and may be recycled within the condensates upon completion of rRNA synthesis. Regulation of the condensate stability will affect NusG functions, potentially offering powerful means to regulate rRNA transcript levels.

### Limitations of the study

Our work relies on tracking labeled NusG molecules to identify NusG diffusive species and uses perturbations to validate our assignments and identify functional NusG pools. However, due to our short tracks (~100 ms), we cannot capture transitions between diffusive states, which can help test our model for NusG utilization (Figure 6). Longer tracks will help visualize full cycles of NusG function, including its entry to anti-termination and transcription-translation coupling. Further, the separation of diffusive species is not absolute, and we cannot exclude the presence of other NusG species with similar diffusive properties, e.g., a small fraction of the S-NusG population may include some NusG complexes with large molecules other than the 30S. Some perturbations may have complex secondary effects since they alter gene expression of several genes; full understanding of all NusG mobility changes will require further studies. Finally, despite our work offering the first view of NusG spatial distribution, more advanced clustering analysis and multi-color imaging will be needed to elucidate the protein stoichiometry and organization inside these transcriptional clusters.

## Star★Methods

### Key Resources Table

**Table:** 

REAGENT or RESOURCE	SOURCE	IDENTIFIER
Antibodies		
Anti-mCherry antibody	abcam	Cat# ab183628; RRID:AB_2650480
Anti-Rabbit IgG (whole molecule)-Peroxidase antibody produced in goat	Merck	Cat# A6154; RRID:AB_258284
Bacterial and virus strains		
MG1655 *F-lambda-ilvG-rfb-50rph-1*	Coli Genetic Stock Centre	CGSC#:7740
DH5α*F- 80dlacZ M15 (lacZYA-argF) U169 recA1 endA1hsdR17(rk-, mk+) phoAsupE44 -thi-1 gyrA96 relA1*	Coli Genetic Stock Centre	CGSC#: 14231
DH5α *λpir endA1 hsdR17 glnV44 (= supE44) thi-1 recA1 gyrA96relA1 φ80dlacA(lacZ)M15 Δ(lacZYA-argF)U169 zdg-232::Tn10 uidA::pir+*	Platt et al.^51^	N/A
PAmCherry-nusG (MG1655 PAmCherry-NusG)	This study	N/A
PAmCherry-nusG ΔnusB FRT-cam-FRT	This study	N/A
MG1655 plac-PAmCherry-nusG	This study	N/A
PAmCherry-NusG WT pRM431	This study	N/A
PAmCherry-nusG nusE-mNeonGreen	This study	N/A
PAmCherry-nusG nusE-mNeonGreen pTRC-sspB	This study	N/A
sfGFP-NusG WT	This study	N/A
sfGFP-NusG ΔnusB	This study	N/A
mNeonGreen-NusG FRT-Kan-FRT	This study	N/A
Chemicals, peptides, and recombinant proteins		
EZ Rich Defined Medium Kit, w/o Methionine	Teknova	M2125
Difco M9 Minimal Salts 5X	SLS LTD.	248510
MEM Amino Acids Solution (50X)-100 mL	Thermo Fisher Scientific	11130036
MEM Vitamin Solution (100X)-100 mL	Thermo Fisher Scientific	11120037
Anti-mCherry antibody	abcam	ab183628
Deposited data		
fluorescence images	This study	https://data.mendeley.com/datasets/7hn4rm94dd/1
Recombinant DNA		
pROD85	Stracy et al.^17^	N/A
pCH101	Mooney et al.^4^	N/A
pNusGPAM do	This study	N/A
pACBSR	Herring et al.^23^	N/A
pRM431	Mooney et al.^4^	N/A
pRM442	Mooney et al.^4^	N/A
pLacUV5 PAmCherry-nusG	This study	N/A
pTRC-sspB	This study	N/A
pHAF-MNG	This study	N/A
pHAF-MNG-nusG	This study	N/A
Software and algorithms		
MATLAB	MathWorks	https://uk.mathworks.com/products/MATLAB.html
ImageJ	Schneider et al.^52^	https://imagej.nih.gov/ij/
Bacseg	Lab software	https://github.com/piedrro/napari-bacseg
MicrobeTracker	Sliusarenko et al.^53^	http://microbetracker.org/
SoftWoRx	Cytiva life sciences	https://download.cytivalifesciences.com/cellanalysis/download_data/softWoRx/7.2.1/SoftWoRx.htm

## Resource Availability

### Lead contact

Further information and requests for resources should be directed to the Lead Contact, Achillefs N. Kapanidis (achillefs.kapanidis@physics.ox.ac.uk).

## Materials availability

All strains generated in this study are available without restriction upon request.

## Experimental Model And Study Participant Details

### Bacterial and virus strains

Plasmids and strains are listed in Table S2. All strains used in the experiments were built in MG1655 background. PAmCherry, sfGFP, mNeonGreen, and degron fusion proteins are expressed from their native chromosome loci. PAmCherry-NusG and sfGFP-NusG were built by gene doctoring. mNeonGreen-NusG, and NusE-degron strains were built using Lambda red recombination. All constructed mutants were subsequently transferred to PAmCherry-NusG or sfGFP-NusG strain by using P1 transduction. Most experiments were run in M9GluVA, which is a supplemented M9 media with MEM amino acids and vitamins. For Richer media experiments, EZ RDM (Teknova) medium was used to get doubling times reminiscent of those obtained in LB but without the autofluorescence that impedes imaging.

## Method Details

### Strain construction

sfGFP-NusG and PAmCherry-NusG fusions were constructed as N-terminal fusions using gene doctoring^23,24^ as previously described by Mooney et al.^25^. Briefly, we used Gibson assembly^54^ to clone either sfGFP or PAmCherry coding sequences followed by a flexible linker sequence (GGSGGGSGA) between the start ATG codon and the second codon, flanked by 1-kb homology to serve as the recombination donor. The mNeonGreen-NusG was constructed using λ red recombination after validating that the NusG fusion with the aforementioned linker is functional. Briefly, the NusG coding sequence was cloned using Gibson assembly^54^ downstream to mNeonGreen (excluding the stop codon) along with the linker in pHAF-MNG. Lambda red oligos with 50-bp homology upstream and downstream from the NusG gene were then used to amplify the fusion, followed by λ red recombination^22^ to replace the wild-type copy with the mNeonGreen version. After validation by sequencing, the construct was moved to fresh background by P1 transduction.

For the NusE-mNeonGreen-degron tag construction, we had to address the fact that NusE is essential and its encoding gene *rpsJ* is the first of 11 genes in a ribosomal protein operon. The DAS+4 degron tag^55^ was introduced using the lambda red system^22^ at the end of *rpsJ*, while introducing only the *kan^R^* sequence and an RBS upstream in a way that the *kan^R^* gene behaves as part of the ribosomal protein operon (rather than a stand-alone insulated cassette).

All strains used in our study are found in Table S2.

### Immunoblotting

Cultures of a strain carrying a genomic copy of PAmCherry-NusG and a strain carrying free PAmCherry under the control of a pBAD promoter were diluted from an overnight culture in LB until the OD_600_ reached 0.2; subsequently, 5 ml were collected and spun down, and the pellet was re-suspended in 100 μl of 1xLaemmli buffer. For the arabinose-inducible strain, 0.2% final arabinose was added to the culture, and was left for 20 min before the cells were pelleted and harvested. The cells were boiled for 10 min at 95°C, and 10 μl were loaded on a pre-cast 4-20% mini Protean gel (Biorad) next to 5 μl of pre-stained protein standard ladder. Once migration was completed, Western blotting was performed using the P3 program of the iBlot system (Thermo Fisher Scientific). The membrane was blocked using TBST+6% skimmed milk, incubated with Anti-mCherry antibody (ab183628-100μl) in 1:2,000 TBST-milk, and left shaking at 4°C overnight. We then incubated the membrane at a ratio 1:5,000 with the secondary antibody of goat anti-rabbit coupled with HRP (A6154-1ML) for 1 hr at room temperature. Finally, the Pierce ECL plus western reagent was added to the membranes according to supplier’s recommendation and imaged using a Typhoon FLA 9500 gel scanner (GE Amersham).

For Western blotting (performed to evaluate the PAmCherry-NusG level in different strains), we prepared the strains using the same culturing conditions used for microscopy. Cells were grown in M9GluVA until OD_600_ ~0.2, and then 10 ml of the culture were pelleted and frozen. For the IPTG induction of PAmCherry-NusG, cells with p*lac* PAmCherry-NusG were induced with 2 mM IPTG for 30 min prior to pelleting (until OD_600_ of ~0.3); an uninduced control was used in parallel. When boiling samples, 50 μl of 1xLaemmli buffer was used per 0.1 OD_600_. After boiling the samples, 20 μl of the sample was loaded on the gel, and the rest of the steps were pursued as above.

### Growth rate measurements

The strain carrying PAmCherry-NusG, and the MG1655 strain were serially diluted in LB 1:1,000, incubated at 37°C in a Clariostar plate reader (GMB) and run overnight with measurements taken every 5 min for up to 24 hours. For doubling time calculations, cells were diluted from an overnight culture in fresh media, with sample readings taken every 30 min until reaching an OD_600_ higher than 1.

### Cell preparation for imaging

Single colonies from a streaked plate of the strains were inoculated in one of three media as necessary in this work: M9GluVA (M9 media supplemented with MEM amino acids, MEM vitamins and 0.2% glucose), M9Glu (M9 media supplemented 0.2% glucose), or RDM medium (1x RDM made from EZ Rich Defined Medium Kit, without Methionine (Teknova M2125) supplemented with 0.2% glucose). Cells were grown overnight at 37°C. Cultures containing plasmids were supplemented with the suitable antibiotics of 100 μg/ml ampicillin, 50 μg/ml kanamycin, or 35 μg/ml chloramphenicol. Overnight cultures were diluted and grown for >2 hrs at 37°C to exponential phase (OD_600_<0.2). Cells were centrifuged and immobilized on 1% low-fluorescence agarose (1613100, Biorad) pads, sandwiched between two glass coverslips (no. 1.5 thickness; prior to use, coverslips were heated to 500°C in a furnace for 1 h to remove any fluorescent background particles). Measurements were performed at 21°C. For treatments with inducers or antibiotics, cells were incubated with either 50 μg/ml rifampicin, or 100 μg/ml chloramphenicol, or 500 μg/ml kasugamycin, 12.5 μg/ml retapamulin, or 2 mM IPTG for 30 min unless otherwise indicated.

### Single-molecule imaging and tracking

All single-molecule tracking PALM and brightfield (BF) images were acquired using a custom-built microscope, equipped with three lasers, a 200 mW 405-nm diode laser (MDL-III-405, CNI, Changchun, China), a 70 mW 473-nm diode laser (Stradus 473, Vortran, Roseville, CA, USA) and a 200 mW diode-pumped solid-state 561-nm laser (561L-COL-PP, Oxxius, Lannion, France). The lasers were modulated using a DAQ system (NI cDAQ-9274 chassis, NI 9263 module; National Instruments, Austin, TX, USA), with the 405-nm and 473-nm lasers modulated directly via analogue voltage commands, and the 561-nm laser using an acousto-optic modulator (Gooch & Housego, Ilminster, Somerset, UK). A custom-built LabVIEW virtual instrument (National Instruments, Austin, TX, USA) software was written for laser modulation. The lasers were coupled into single mode optical fibers, collimated, reflected by a multi-bandpass optical filter (69013m, Chroma Technology Corp, Bellows Falls, VT, USA), and focused by an achromatic doublet lens (AC508-300-A, ThorLabs, Newton, New Jersey, USA) onto a single point on the back focal plane of a 100x NA1.40 oil immersion microscope objective (UPlanSApo, Olympus). Exit angle modulation enabled sample illumination in epifluorescence, variable angle epifluorescence microscopy (VAEM) and total internal reflection (TIR) modes – for our cellular imaging, only VAEM configuration was employed. BF images were illuminated using a white LED light source (CoolLED pE-100). Collected light was passed back through the multi-notch filter with transmission windows at 439±15 nm, 521±17 nm, and 605±25 nm, with an achromatic doublet lens (AC508300-A, ThorLabs, Newton, New Jersey, USA) forming an image on an EMCCD camera (iXon 897 Ultra, Andor Technology Ltd, Belfast, UK). Image acquisition was performed using the software package Andor SOLIS (Andor Technology, Belfast, UK). Tracking PALM comprised 561-nm excitation at ~340 W/cm^2^, and 405-nm excitation at 0-1 W/cm^2^; imaging parameters involved a pre-amplified gain of 1, a memory parameter of 1, and 10-ms exposures over 30,000 frame recording.

### SIM imaging

For 3D imaging, 3D-Structural Illumination Microscopy (3D-SIM) was performed as described in^17,56^ with minor adjustments. Briefly, imaging was performed using a Deltavision OMX-SR microscopy system (GE Healthcare) equipped with four laser lines (405, 488, 568 and 640 nm), pco.edge 4.4 sCMOS cameras (PCO) and a 60x oil-immersion objective (Olympus PlanApo 1.42 NA). An area of 512x512 pixels was used to acquire a stack of 125-nm sections to generate a total of 2-3 μm thickness. Each z section results from a striped illumination pattern rotated to the three angles (−60°, 0°, +60°) and shifted in five phase steps. NusG-sfGFP was excited using 10% of the 488-nm laser power and imaged using 10-ms exposures, whereas the DAPI stain was excited using 20% of the 405-nm laser power and imaged using 20-ms exposures. The image stacks were 3D-reconstructed using Deltavision softWoRx 7.2.0 software with a Wiener filter of 0.003 using wavelength-specific experimentally determined OTF functions. Average intensity and 3D projections of 3D-SIM images were generated using ImageJ to generate the two-color 3D-SIM images. Notably, SIM signals cannot be used quantitatively at the moment^57^; we thus discuss our results only qualitatively.

### Image and data analysis for PALM imaging

Image and data analysis were performed as we previously described.^17^ Briefly, single-molecule localizations in cells were performed using custom in-house tracking software (“StormTracker”). Initial cell segmentation using a segmentation algorithm (based on Mask R-CNN^58^and training on bright-field images) was followed by mesh refinement via Microbetracker.^53^ Custom in-house software (“LoColi”) was used to filter localizations using a segmentation mask and generate tracks that were used to calculate diffusion coefficients D* for each track. D* histograms were generated from the compilation of triplicate datasets and were fitted to gamma distributions^17^. Heatmaps for the intracellular locations of each localization in the selected tracks were computed for both different diffusive populations (using a specified D* threshold to examine tracks of different mobility), and normalized across cell length. All the results from the fitting of different D* distributions to a number of diffusive species are summarized in Table S1. To calculate fitting errors in population amplitudes, we carried bootstrapping analysis essentially as in Stracy et al.^20^. Briefly, we run bootstrap resampling with replacement of individual tracks (100 times); fitted the resulting D* distribution and recovered the population amplitudes for each run; and calculated the standard deviation for each population amplitude for the main diffusive species (VS-, S-, and F-NusG). In all cases, we get a standard deviation of <1% in the population amplitude (Figure S1), which is a very small error.

To visualize the spatial distribution of different diffusive species, we plotted the spatial distribution of tracks that are within specific D* threshold values, which were selected to visualize preferentially a specific diffusive species. This simple approach does not yield 100% “pure” species, since the heat maps carry tracks of the remaining one or two diffusive species. To estimate the fraction of tracks corresponding to the main diffusive species captured by D* thresholding (as in Figures 1C and 4C), we used the areas under the fitted curves for all diffusive species (3 species in Figure 1C, and 2 species in Figure 4C) for all the ranges defined by thresholding; we reported these fractions for each main diffusive species in Figures 1C and 4C.

### Estimation of the fraction of time NusG spends in transient DNA binding

The fraction of time that a DNA-binding protein binds non-specifically to the chromosome can be estimated by a simplified version of Elf et al.^59^ using D_intact_ = (1-f_ns_)* D_free_, where D_intact_ and D_free_ are the accurate protein diffusion coefficients in cells with intact nucleoids, and in cells in the absence of DNA binding, respectively, and f_ns_ is the fraction of time that the protein binds non-specifically to the DNA.

## Quantification And Statistical Analysis

Experimental statistics and quantification values can be found in the figure legends and Table S1. Values include number of cells, number of trajectories, average track length, population fractions, and population allocation error percentages. Multiple fields of view were collected per experiment to collect sufficient numbers of cells for adequate statistical sampling. Experimental repeats for each condition were performed to define the reproducibility of the results.

## Supplementary Material

Supplemental information can be found online at https://doi.org/10.1016/j.molcel.2024.01.025.

supplementary

## Data Availability

Fluorescence images of cells are deposited on Mendeley (Mendeley Data: https://data.mendeley.com/datasets/7hn4rm94dd/1). This paper does not report original code. Any additional information required to reanalyze the data reported in this paper is available from the lead contact upon request.

## In brief

El Sayyed et al. used single-molecule imaging in live *E. coli* cells to identify *rrn* anti-termination (with NusG in transcriptional condensates) and transcription-translation coupling as the main processes occupying NusG *in vivo*. The work also revealed previously unknown NusG interactions with the free 30S ribosomal subunit and the chromosome.

## Highlights

- There is very little free NusG in the cell, with most NusG forming large complexes
- Any free NusG interacts non-specifically with the chromosome >50% of the time
- Most NusG regulates anti-termination on *rrn* genes, where NusG is found in condensates
- NusG binds to free 30S subunits, helping initiate transcription-translation coupling
