# Supplementary material for: Single-molecule tracking reveals the functional allocation, in vivo interactions, and spatial organization of universal transcription factor NusG
Source: Mol Cell. Author manuscript; Available in PMC 2025 Oct 24. (PMC7618293; doi:10.1016/j.molcel.2024.01.025)
Supplement: supplementary [file EMS209512-supplement-supplementary.pdf]

Molecular Cell, Volume *84*

Supplemental information

Single-molecule tracking reveals the functional allocation, *in vivo* interactions, and spatial organization of universal transcription factor NusG

Hafez El Sayyed, Oliver J. Pambos, Mathew Stracy, Max E. Gottesman, and Achillefs N. Kapanidis

SUPPLEMENTARY INFORMATION

Single-molecule tracking reveals the functional allocation, *in vivo* interactions and spatial organization of universal transcription factor NusG

Hafez El Sayyed[1,2,*], Oliver J. Pambos[1,2], Mathew Stracy[3], Max E. Gottesman[4], and Achillefs N. Kapanidis[1,2,*]

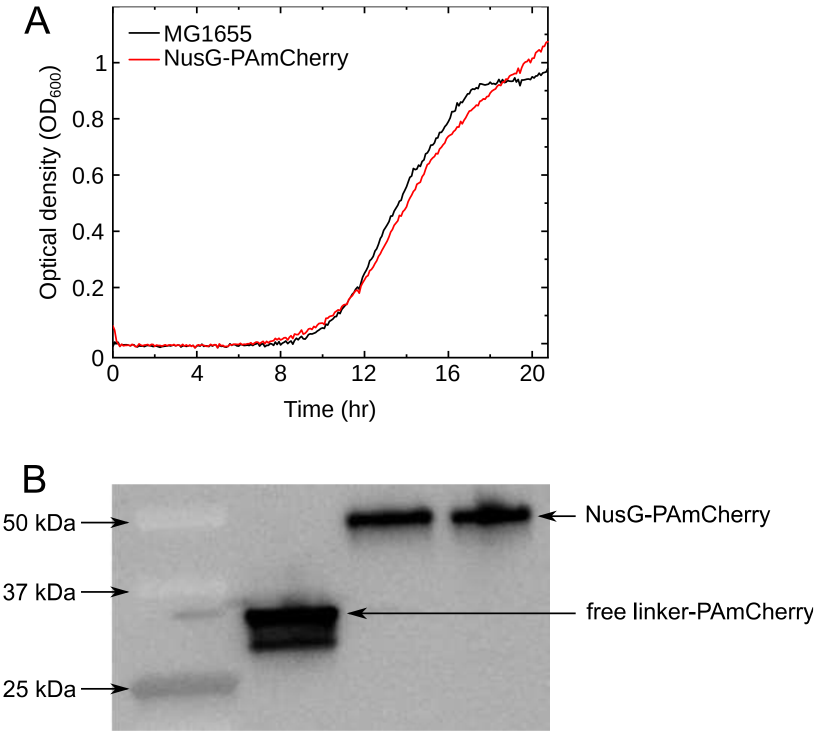

Figure S1. **Verification of the PAmCherry-NusG fusion**, related to Figure 1.

A. Growth curve for wild type MG1655 (black) and the PAmCherry-NusG fusion (red) grown in LB overnight, then diluted 1:1,000 in a 96-well plate in triplicates. Readings were taken every 4 min for 24 hrs.

B. Western blot to validate that the PAmCherry-NusG fusion is expressed intact. Left, molecular weight marker; middle, lysate from a control strain DH5α carrying an arabinose-inducible plasmid expressing PAmCherry plus a linker; right, lysate from the genome-encoded PAmCherry-NusG fusion strain. Lysates were probed using the anti-mCherry antibody as described in *Methods*. The intact nature of PAmCherry-NusG is indicated by the band appearing at the combined size of both proteins in comparison to the free PAmCherry fusion alone.

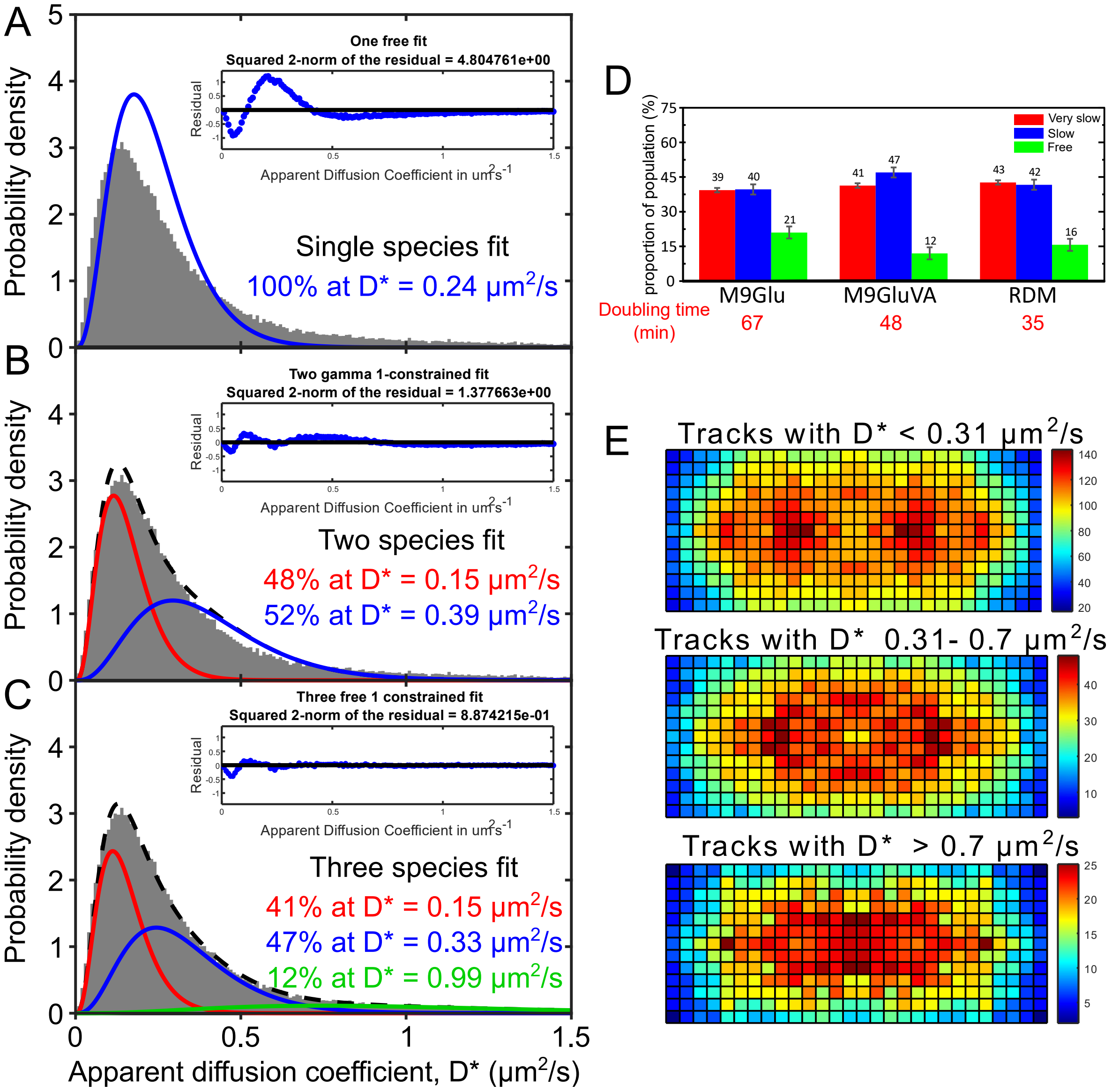

Figure S2. **Sorting of NusG D* distribution into different diffusive species, and visualizing their spatial distribution,** related to Figure 1.

A. Single-species fit to the D* distribution of NusG. Inset, plot of fit residuals as a means to assess fit quality, along with squared 2-norm of residuals (~4.8).

B. As in A, but for a two-species unconstrained fit. The residuals show a substantial better fit (squared 2-norm of ~1.38).

C. As in A, but for a three-species fit. The residuals show an improvement of the 2-species fit (more evenly distributed residuals, and a squared 2-norm of ~0.89).

D. The fractions of the NusG D* distribution that correspond to the VS-NusG, S-NusG, and F-NusG species for the three growth media used in this study. Error bars represent the standard error between 3 different datasets in each condition.

E. Spatial distribution heatmaps of NusG tracks for 190 cells with lengths of ~1-2 μm, having ~1 nucleoid. Top: a heatmap for molecules with D* < 0.31 μm²/s, representing mainly the VS-NusG species. Middle: a heatmap for molecules with D* between 0.31 and 0.7 μm²/s, representing mainly the S-NusG species. Bottom: a heatmap for molecules with D* > 0.7 μm²/s, representing mainly the F-NusG species.

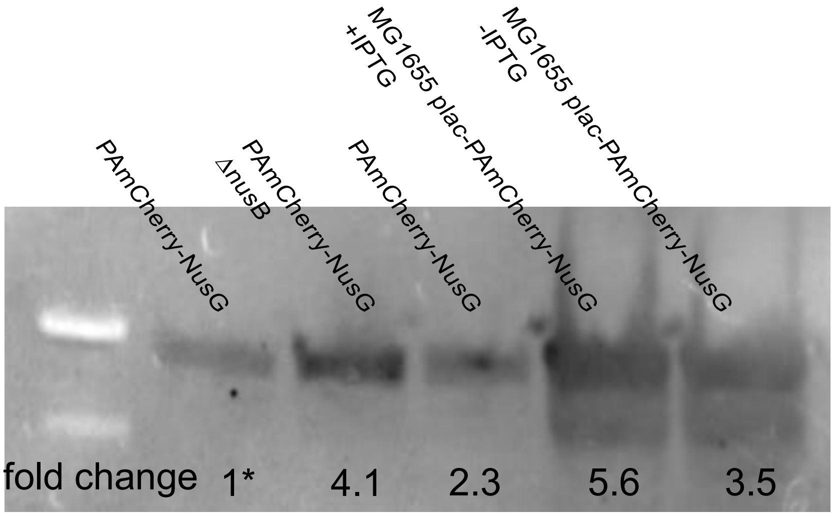

Figure S3. Quantification of the PAmCherry-NusG levels in different strain backgrounds, related to Figures 1 and 3.

Western blot to quantify the PAmCherry-NusG expression level in the WT genomic background versus in the Δ*nusB* background, and in PAmCherry-NusG overexpression strains. Insets, intensity signals relative to PAmCherry-NusG (2nd lane, depicted as 1*) based on densitometry analysis of the bands; the second PAmCherry-NusG band (4th lane) had a higher value due to an impurity in the center of the band.

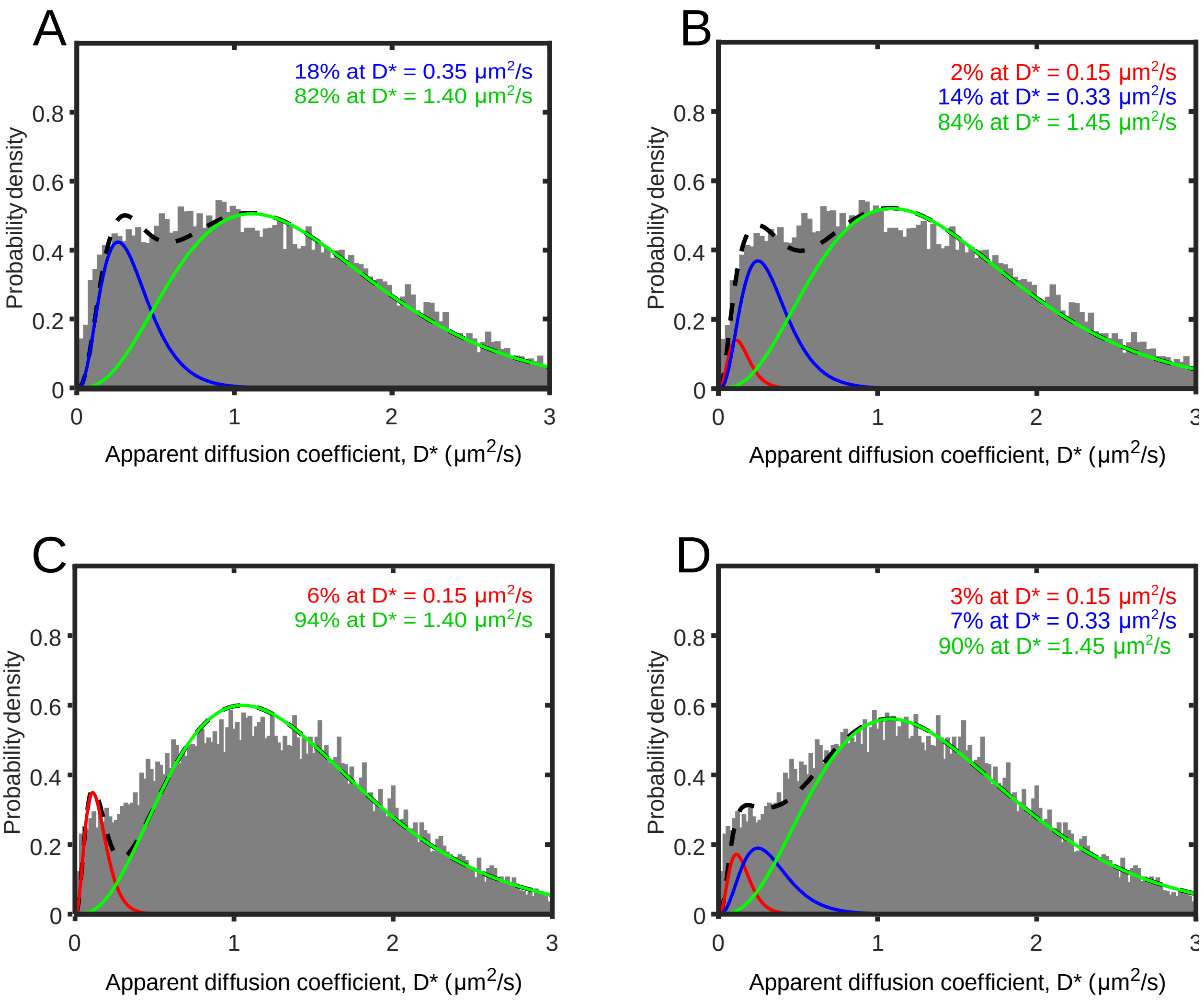

Figure S4. Comparison of 2-species vs 3-species fit for the NusG D* distribution for the Rif-treatment and NusE degron experiments, related to Figures 2 and 4.

A. Two-species fit of the D* distribution for NusG in Rif-treated cells (as in Figure 2C).

B. Three-species fit of the D* distribution in panel A, using the D* of the three populations observed in the untreated data (Fig. 1B). Upon fitting three species, VS-NusG accounted for only 2% of the tracks, whereas the S-NusG fraction only modestly decreased relative to its value in the 2-species fit; we conclude that the 2-species fit is sufficient for describing the data.

C-D. A similar comparison between 2-species and 3-species fits for the NusG D* distribution from the NusE-degron experiment from Fig. 4B.

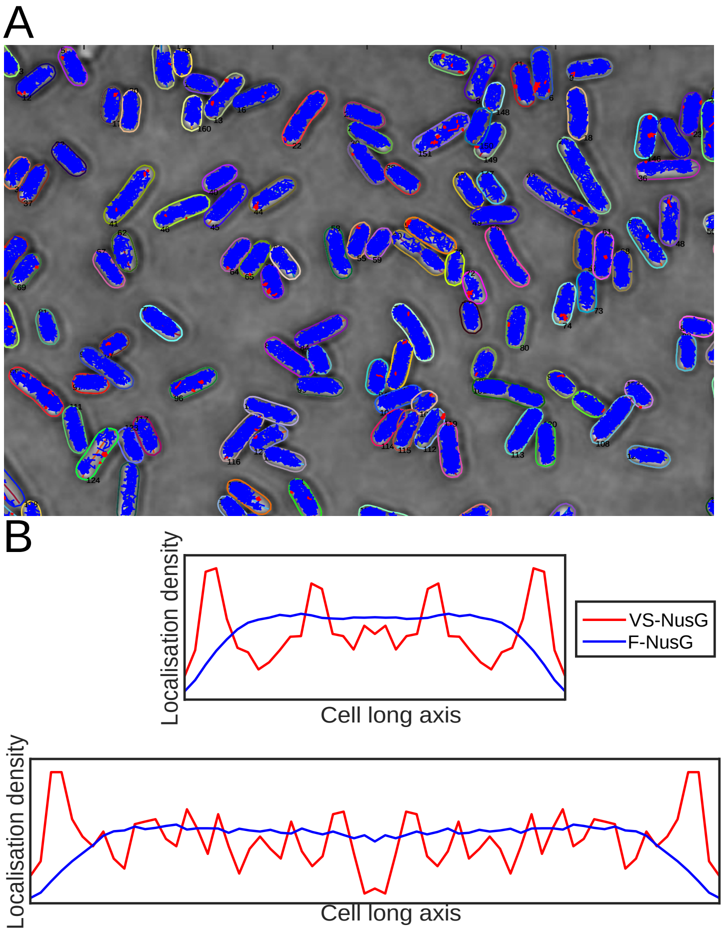

Figure S5. Spatial distribution of the VS-NusG population in the NusE degron experiments, related to Figure 4.

A. Representative field of view shows PAmCherry-NusG tracks upon NusE degradation. Blue tracks: F-NusG tracks; since there is a very large number of tracks per cell, the tracks overlap and the entire cell is coloured blue. Red tracks: VS-NusG tracks. Tracks are very few and tend to localize at locations close to the poles, and in general, in locations excluded from the nucleoid.

B. Long-axis projections of species of different mobility in the NusE degron experiments. Track distribution in small cells containing a single nucleoid (top panel) and in large cells containing two nucleoids bottom panel).

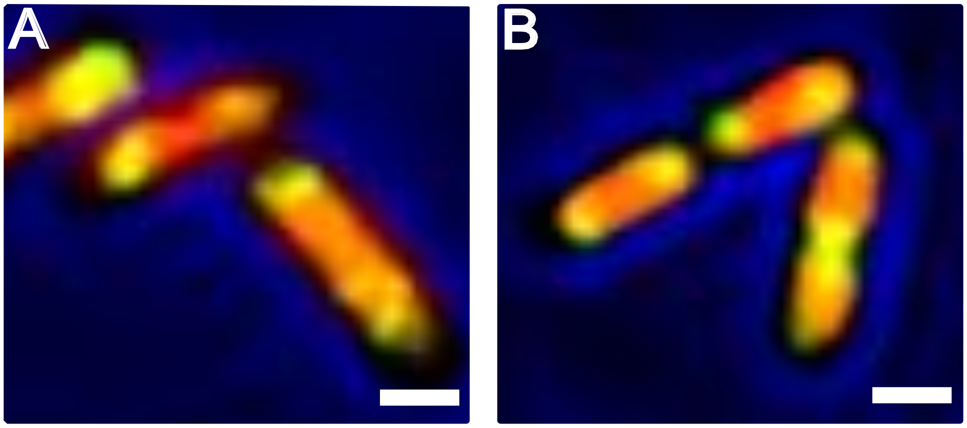

Figure S6. NusG spatial distribution after translation initiation inhibition by kasugamycin and retapamulin, related to Figure 4.

A-B. Fluorescence microscopy images of mNeonGreen-NusG cells treated with 500 µg/ml kasugamycin (panel **A**) or with 12.5 µg/ml retapamulin (which targets the 50S ribosomal subunit) for 30 min (panel **B**). Cells were grown in M9GluVA treated with both 500 nM SYTOX Orange (S11368, Thermo-Fisher) and either kasugamycin or retapamulin for 30 min. Cells were then washed with media (containing the antibiotics) to remove unbound SYTOX orange, and were then imaged using 100-ms exposures. Images show mNeonGreen-NusG (green), brightfield (blue), and SYTOX orange labelling the DNA (red). Scale bars, 1 µm. Both treatments result in NusG clusters that tend to localize close to the poles.

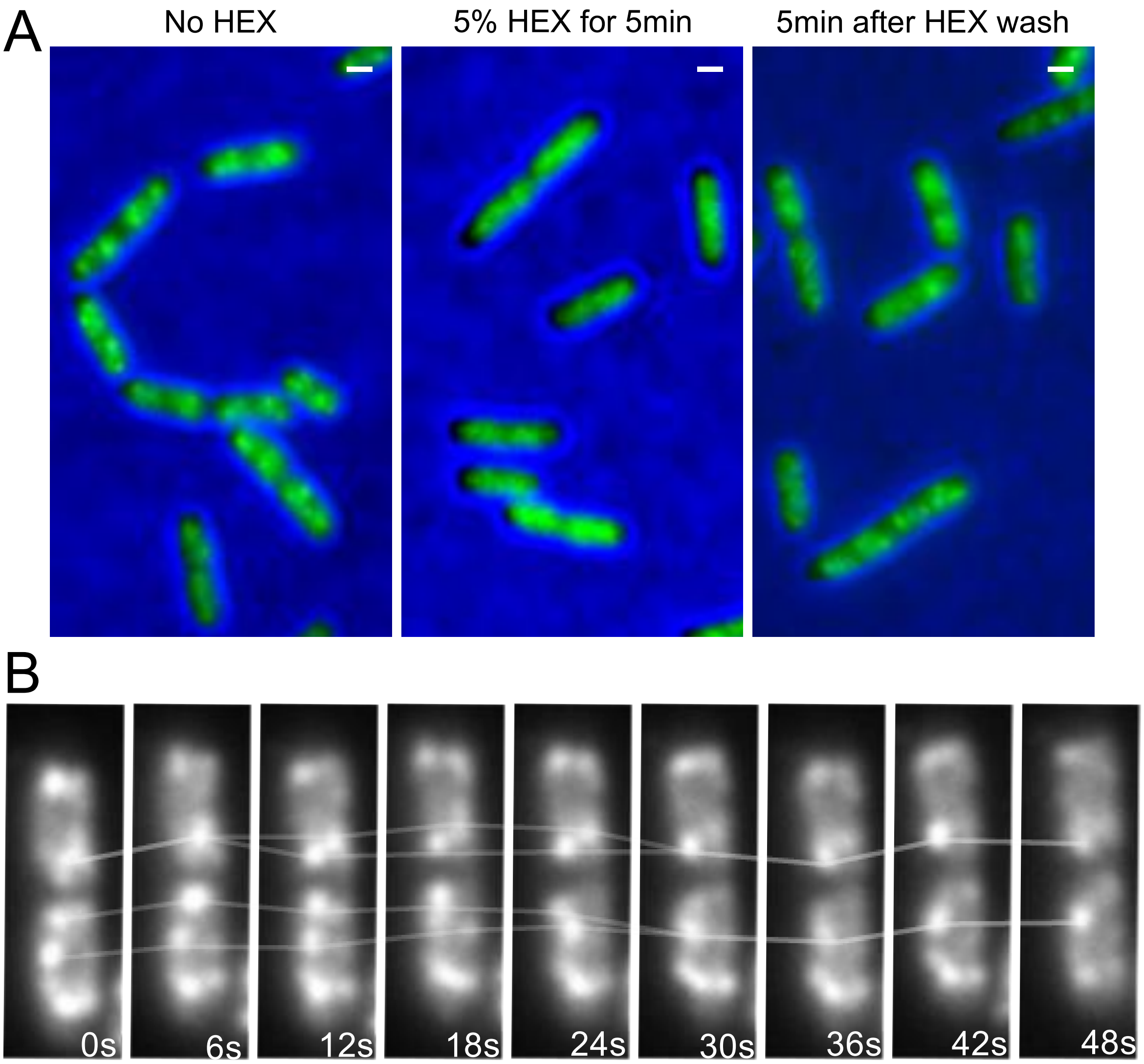

Figure S7. Measurement of the dynamic nature of NusG clusters, related to Figure 5**.**

A. mNeonGreen-NusG was grown in M9GluVA media until OD600 ~ 0.2 at 37˚C. The samples were fixed at three instances: prior to HEX addition ("NO HEX" sample); after 5 min of 5% HEX treatment; and 5 min after samples were washed with fresh media without HEX for 5 min. Samples were imaged at 200-ms exposures. Scale bars, 1 µm.

B. Time lapse microscopy of mNeonGreen-NusG grown in Rich Defined Media (EZ) and imaged at 37˚C. Ten 100-ms images were acquired and averaged every 5s. Bleaching was corrected in ImageJ using a single-exponential fitting method. Images show examples of a cluster fusion event (top) and cluster splitting event (bottom), although such events are infrequent.

Table S1

	population in %				
Experiment	VS-NusG	S-NusG	VS/S	F-NusG	Figure
no treatment	41 (0.4)	47 (0.36)	-	12 (0.2)	1B
unlabelled NusG overepxression	-	-	8 (0.5)	92 (0.4)	2A
PAmCherry-NusG overexpression	-	-	4 (0.36)	96 (0.36)	2B
Rifampicin treatment	-	18 (0.5)	-	82 (0.5)	2C
ΔnusB	18 (0.38)	28 (0.39)	-	54 (0.6)	3B
NusE degradation	6 (0.3)	-	-	94 (0.4)	4B
Kasugamycin treatment	28 (0.7)	55 (0.8)	-	17 (0.3)	4E
Retapamulin treatment	35 (0.59)	51 (0.68)	-	14 (0.5)	4F
1,6-Hexanediol treatment	17 (0.27)	27 (0.43)	-	56 (0.5)	5B

Table S2

Name	aim	genotype	reference
Strains			
MG1655 wild type		wild type	Lab strain
DH5a		F−endA1 hsdR17 supE44 thi-1 recA1 gyrA relA1 ΔlacU169 80lacZΔM15	Lab strain
DH5a Pir	pir+ to propagate r6K plasmids	endA1 hsdR17 glnV44 (= supE44) thi-1 recA1 gyrA96 relA1 φ80dlacΔ(lacZ)M15 Δ(lacZYA-argF)U169 zdg-232::Tn10 uidA::pir+	Platt et al., Plasmid 43 (2000), 12-23 [PMID: 10610816]
PAmCherry-nusG WT	NusG N-terminal PAmcherry fusion	MG1655 PAmCherry::nusG	this work
PAmCherry-nusG ΔnusB	To test effect of nusB deletion	MG1655 PAmCherry::nusG ΔnusB:camR	this work
MG1655 plac-PAmCherry-nusG	Test PAmCherry free diffusion	wild type	this work
PAmCherry-NusG WT pRM431	overexpression of full nusG to compete with PAmCherry-NusG	MG1655 PAmCherry::nusG	this work
PAmCherry-nusG nusE-mNeonGreen	nusE degradation in vivo	MG1655 nusG::PAmCherry ΔsspB:frt nusE::DAS4 degron :kanR	this work
PAmCherry-nusG nusE-mNeonGreen pTRC-sspB	With the plasmid that induces degradation	MG1655 nusG::PAmCherry ΔsspB:frt nusE::DAS4 degron :kanR	this work
sfGFP-NusG WT	to do ensemble and SIM experiemnts	MG1655 sfGFP::nusG	this work
sfGFP-NusG ΔnusB	to do ensemble and SIM experiemnts	MG1655 sfGFP::nusG ΔnusB:camR	this work
mNeonGreen-NusG	Better fluorophore for imaging time-lapses	Frt Kan frt MG1655 mNeonGreen::nusG	this work
plasmids			
pROD85	carrying PAmCherry1 gene preceeded by an 11 aa linker and has frt:kan:frt cassette	kanR	a gift from the Sherrat lab
pCH101		kanR	Mooney et al, 2009
pNusGPAM do	derivative of pCH101 to intoduce PAmCherry in NusG by gene doctoring	kanR	this work
pACBSR	plasmid used for gene doctoring	camR	Herring et al, 2003
pRM431	overexpression of full NusG	ampR	Mooney et al, 2009
pRM442	overexpression of NusG-NTD	ampR	Mooney et al, 2010
pLacUV5 PAmCherry-nusG	overexpression of NusG-Pamcherry in wt strains	camR	this work
pTRC-sspB	inducible degron degradation, a derivative of pRM431	ampR	this work
pHAF-MNG	Template for mNeonGreen fusion integration	kanR	This work
pHAF-MNG-nusG	Template made to insert mNeonGreen at NusG N- [illegible]	kanR	This work

Supplemental information References:

1. Stracy, M., Lesterlin, C., De Leon, F.G., Uphoff, S., Zawadzki, P., Kapanidis, A.N., and Ha, T. (2015). Live-cell superresolution microscopy reveals the organization of RNA polymerase in the bacterial nucleoid. Proc. Natl. Acad. Sci. U. S. A. *112*, E4390–E4399. 10.1073/pnas.1507592112.

2. Herring, C.D., Glasner, J.D., and Blattner, F.R. (2003). Gene replacement without selection: Regulated suppression of amber mutations in Escherichia coli. Gene *311*, 153–163. 10.1016/S0378-1119(03)00585-7.

3. Mooney, R.A., Schweimer, K., Rösch, P., Gottesman, M., and Landick, R. (2009). Two Structurally Independent Domains of E. coli NusG Create Regulatory Plasticity via Distinct Interactions with RNA Polymerase and Regulators. J. Mol. Biol. *391*, 341–358. 10.1016/j.jmb.2009.05.078.

4. Platt, R., Drescher, C., Park, S.K., and Phillips, G.J. (2000). Genetic system for reversible integration of DNA constructs and lacZ gene fusions into the Escherichia coli chromosome. Plasmid *43*, 12–23. 10.1006/plas.1999.1433.
